# Skewness and Staging: Does the Floor Effect Induce Bias in Multilevel AR(1) Models?

**DOI:** 10.1080/00273171.2023.2254769

**Published:** 2023-12-31

**Authors:** MohammadHossein M. Haqiqatkhah, Oisín Ryan, Ellen L. Hamaker

**Affiliations:** a Department of Methodology and Statistics, Faculty of Social and Behavioural Sciences, Utrecht University, Utrecht, The Netherlands; b Julius Center for Health Sciences and Primary Care, University Medical Center, Utrecht University, Utrecht, The Netherlands

**Keywords:** Floor effect, experience sampling, emotional inertia, staging effect, non-Gaussian time series

## Abstract

Multilevel autoregressive models are popular choices for the analysis of intensive longitudinal data in psychology. Empirical studies have found a positive correlation between autoregressive parameters of affective time series and the between-person measures of psychopathology, a phenomenon known as the *staging effect*. However, it has been argued that such findings may represent a statistical artifact: Although common models assume normal error distributions, empirical data (for instance, measurements of negative affect among healthy individuals) often exhibit the *floor effect*, that is response distributions with high *skewness*, low mean, and low variability. In this paper, we investigated whether—and to what extent—the floor effect leads to erroneous conclusions by means of a simulation study. We describe three dynamic models which have meaningful substantive interpretations and can produce floor-effect data. We simulate multilevel data from these models, varying skewness independent of individuals’ autoregressive parameters, while also varying the number of time points and cases. Analyzing these data with the standard multilevel AR(1) model we found that positive bias only occurs when modeling with random residual variance, whereas modeling with fixed residual variance leads to negative bias. We discuss the implications of our study for data collection and modeling choices

## Introduction

In the past two decades, the collection of intensive longitudinal data based on techniques like the experience sampling method, ambulatory assessments, and daily diaries has become increasingly popular in psychological research (Bolger & Laurenceau, 2013). To study the dynamics in these data, multilevel versions of the first-order autoregressive (AR(1)) model and the multivariate version, known as the first-order vector autoregressive (VAR(1)) model, are often used (Asparouhov et al., 2018; Bringmann et al., 2013; Epskamp et al., 2018). In these models, the current observation is regressed on itself at the preceding measurement occasion through the autoregressive or *inertia* parameter (Cook et al., 1995; Koval & Kuppens, 2012; Kuppens et al., 2010; Suls et al., 1998). Research has shown that higher inertia in affective measures tends to be associated with higher levels of neuroticism (Koval et al., 2015;; Suls et al., 1998) and depression (Houben & Kuppens, 2020), and lower levels of psychological well-being (Ebner-Priemer et al., 2015; Houben et al., 2015). Other research has shown that the cross-lagged parameters between symptoms measured at subsequent occasions are positively correlated to severity of psychopathology (see, e.g., Bringmann et al., 2013), and may distinguish between stages of mental disorders, which has been referred to as the *staging effect* (Wigman et al., 2013). Together, these results have been interpreted to mean that stronger lagged relations are a sign of maladaptive regulation: Higher autoregressions are thought to reflect emotional rigidity and imply a longer recovery time, whereas higher cross-regressions may imply a faster flow of activation through a network of emotions or symptoms (Bringmann et al., 2022, Bringmann et al., 2015, Bringmann et al., 2016).

Recently, however, concerns have surfaced about the mismatch between the multilevel models that are typically used in this line of research, and the distributional features of the empirical data. The multilevel models are typically based on the assumption that all the data are normally distributed. However, for variables such as psychological symptoms or negative affect, individuals with relatively low average scores often display the *floor effect*, characterized by less variability and more skewness, while individuals with higher average scores tend to have wider and more symmetric distributions (Falcaro et al., 2013; Peeters et al., 2006; von Klipstein et al., 2022). Ignoring these distributional differences may have serious consequences for studying staging, according to Terluin et al. (2016): They argued that lower variability will lead to underestimation of the autoregressive parameter, and since lower variability is associated with lower means, this would imply that the staging effect may be nothing but a statistical artifact, resulting from a failure to account for individual differences in distributions. Although Terluin et al. (2016) did not provide rigorous analytical support for this claim,^1^ they did present some empirical evidence, based on reanalyzing a dataset that had been used to show staging before. When using a multilevel model based on the inverse Gaussian regression model, which is often proposed to handle right-skewed data, they found that there was no evidence for staging anymore.

The results of Terluin et al. (2016) have spurred widespread concerns among the inertia and psychological networks researchers (see, e.g., Forbes et al., 2017; McNally, 2021; Rodebaugh et al., 2018; Wright & Zimmermann, 2019). However, it is not clear yet why the autoregression for individuals with a stronger floor effect would be underestimated—a premise to conclude that the staging effect is an artifact. Moreover, Terluin et al. (2016) did not discuss whether the inverse Gaussian regression model could be considered a plausible data-generating mechanism underlying skewed empirical time series. This makes it hard—if not impossible—to tell whether their approach provides valid results, or that it actually overcorrects for the floor effect in the data (Schmidt & Finan, 2018) and in doing so fails to uncover the staging effect that is actually present. The goal of the current paper is therefore to: (a) present alternative data-generating models, for which we provide substantive explanations, and that can produce different degrees of skewness in the autocorrelated time series of different individuals; and (b) use these data-generating models to simulate data with the floor effect and without the staging effect, to determine whether using an analysis technique that ignores individual differences in the floor effect erroneously detects a staging effect.

This paper is organized as follows. In the first section, we begin with the single-person AR(1) model, and show how this is a building block in the multilevel AR(1) model. Moreover, we present empirical data that show how the assumptions of normally distributed scores within and between individuals can be violated in practice. In the second section, we present three alternative time series models that represent distinct assumptions regarding the nature of the processes that may explain various forms of the floor effect surfacing in empirical data collected using different scales. In these models, the mean—and consequently, the variance and skewness—of the processes can be specified independently from their autoregression. Subsequently, in the third section, we perform a simulation study where we use the proposed alternative time series models to generate multilevel data with lag-1 autocorrelation. We analyze the data using the multilevel AR(1) model to explore the conditions under which neglecting the floor effect might result in mistakenly detecting a staging effect, and investigate the extent to which this may occur. Finally, we conclude the paper by reflecting on the implications of our findings for empirical researchers.

## Background

In this section, we provide a brief introduction to the AR(1) model and the multilevel AR(1) model. Subsequently, we discuss the main assumptions that underlie the multilevel AR(1) model, which are violated when there is a floor effect. Furthermore, we demonstrate the presence of the floor effect in an empirical dataset based on self-reported measures of affect.

### The first-order autoregressive (AR(1)) model

The AR(1) model is a popular choice for the analysis of univariate time series data (Gottman, 1981; Shumway & Stoffer, 2017), in which the variable at the current measurement occasion *X_t_* is regressed on that same variable at the preceding measurement occasion *X_t_*_–1_ (Krzysztofowicz & Evans, 2008). The AR(1) model has also gained popularity in psychological research (Hamaker & Dolan, 2009; Koval et al., 2021), particularly due to its substantive appeal: Many psychological time series, for instance, of emotions, are persistent, self-predictable, and manifest a relative resistance to change (Frijda, 1992; Koval et al., 2021) as well as considerable autocorrelation (Gottman et al., 1969; Huba et al., 1976).

The AR(1) model can be written as

(1) Xt=c+ϕXt−1+ϵt,

where *c* represents an intercept or constant term, ϕ is called the autoregressive parameter, and *ϵ_t_* represents a residual term or random perturbation called the *innovation*, which is typically assumed to be normally distributed with $\epsilon t∼N(0,\sigma_{\epsilon }^{2}).$ In psychological settings, where the variable *X_t_* may represent, for instance repeated measures of momentary distress, the parameter ϕ is often referred to as *inertia*, because the closer it is to 1, the more carry-over there is of current distress to distress at the next time point (Koval et al., 2021). The predictable part is sometimes called the *conditional expectation* and is formed by

(2) $$\begin{array}{c} E[Xt|Xt-1]=c+\phi Xt-1. \end{array}$$

The innovation term represents the random or unpredictable part of the model. In psychology, the variance of the innovations ($\sigma_{\epsilon }^{2}$) is interpreted as capturing the actual variability of perturbations as well as the sensitivity of the person to external perturbations (Jongerling et al., 2015).

A core assumption of the AR(1) model is that the process under investigation is *stationary*, which means that the mean and the variance do not change over time. To ensure stationarity, the absolute value of ϕ should be smaller than 1, that is, |ϕ|<1 (Box et al., 2016). Under this assumption the long-run mean of the AR(1) process, E[Xt]=μ, is determined by both the intercept and the autoregressive coefficient, through

(3) $$\begin{array}{c} \mu =\frac{c}{1-\phi }. \end{array}$$

The distribution of *X_t_* values, in the long-run, is called the *marginal*, or *stationary*, *distribution*. The marginal distribution of the AR(1) process is Gaussian (or normal) with mean *μ* and a variance that is a function of the autoregressive parameter and the innovation variance, that is

(4) $$Xt∼N(\frac{c}{1-\phi },\frac{\sigma_{\epsilon }^{2}}{1-\phi^{2}}).$$

This implies that the variability of a person’s distress is not only determined by variability of the external events (or the person’s sensitivity to them), but also the individual’s inertia. Furthermore, as the normal distribution is symmetrical, the AR(1) model has a skewness of *γ* = 0.

One final way in which we can characterize the AR(1) process is by defining how current observations (*X_t_*) correlate to observations in the past (Xt−l,Xt−2,…). This is given by the autocorrelation function (ACF), which is the correlation of the sequence with its lagged versions. For the AR(1) model of Equation 1, the autocorrelation for lag l≥0 is given by

(5) $$\rho (l)=\phi^{l},$$

such that as *l* gets larger, ρ(l) gets smaller exponentially. These properties of the AR(1) model are derived in the Supplemental Materials.^2^

**Figure 1.**
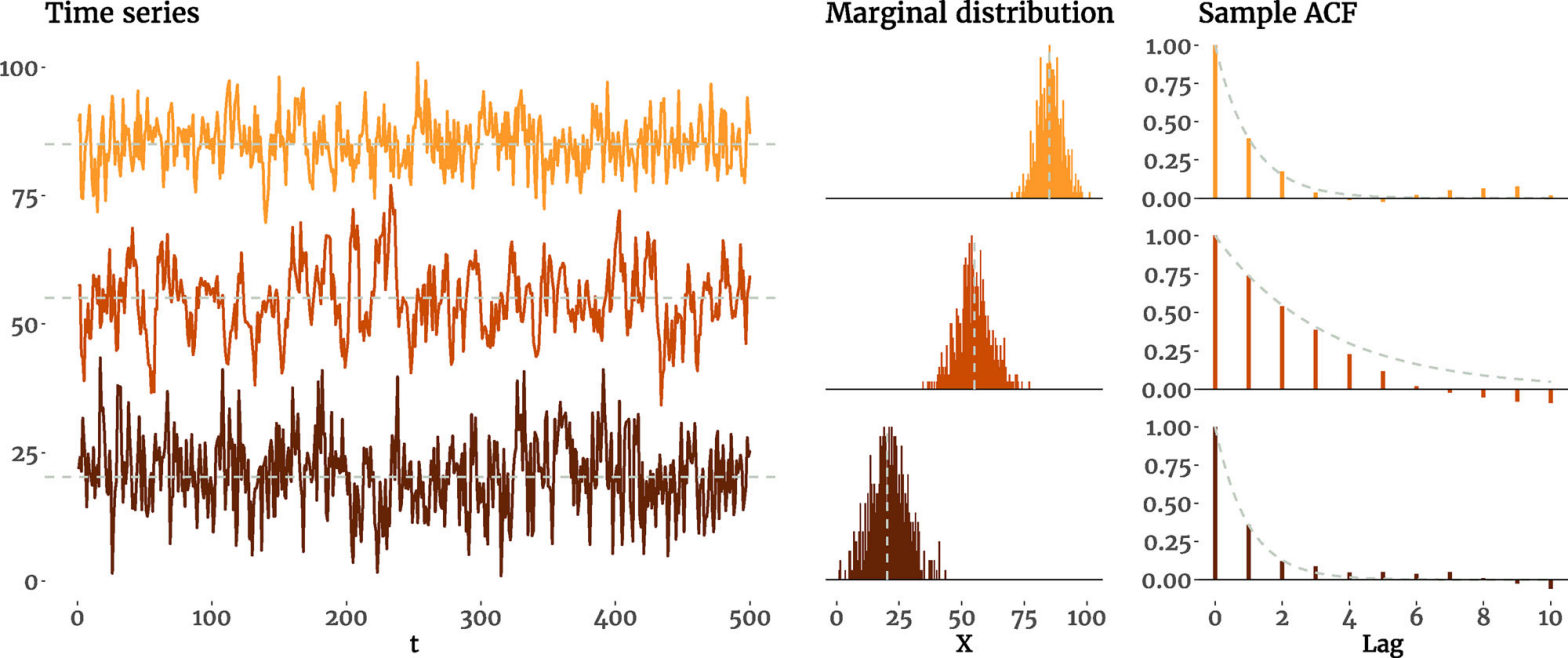
Time series plots, marginal distributions, and the sample ACF of three simulated AR(1) processes with different model-implied means (*μ*), residual variances ($\sigma_{\epsilon }^{2}$), autoregressive parameters (ϕ). In the first process, we have *μ* = 85, $\sigma_{\epsilon }^{2}=20,$ and ϕ=0.4, which produce a distribution with marginal variance $\sigma^{2}=23.69$ and marginal skewness γ=0.01; the second process has *μ* = 55, $\sigma_{\epsilon }^{2}=20,$ and ϕ=0.8, resulting in $\sigma^{2}=55.37$ and *γ* = 0; and the last process has *μ* = 20, $\sigma_{\epsilon }^{2}=47,$ and ϕ=0.4, leading to $\sigma^{2}=55.51$ and γ=−0.01. The dashed lines in the first two columns mark the mean, and in the right column trace the exponential decay of the theoretical ACF.

In Figure 1 we show three simulated AR(1) processes with different parameters; the left column shows the time series generated by the model, the middle column shows the marginal distributions of the time series, and the right column includes the sample ACF of each process. We can see that processes may differ in some properties while being similar in others; comparing the second and third processes, we see that, for instance, two processes can have similar marginal variance, while being very different in all other aspects (the mean, residual variance and autoregressive parameters). In the remainder of the paper we will use these properties to compare the alternative data-generating models to the AR(1) model.

### The multilevel AR(1) model

In psychological research, the multilevel AR(1) model has been a popular choice for the analysis of intensive longitudinal data from multiple individuals (Koval et al., 2015; Kuppens et al., 2010; Rovine & Walls, 2006). While there are various ways to specify this model (Jongerling et al., 2015), here we will start with decomposing the observed variable of individual *i* at occasion *t* (i.e., Xi,t) into a person-specific mean *μ_i_* that can be interpreted as the person’s home-base or equilibrium score, and a temporal, person-specific deviation from this mean, which we denote by $\tilde{X}i,t.$ Specifically, we can write

(6) $$\begin{array}{cc} Xi,t & =\mu i+\tilde{X}i,t. \end{array}$$

The temporal, person-specific deviation from their mean, $\tilde{X}i,t,$ is used in the level 1 or within-person equation of the multilevel model, which is modeled with the first-order autoregressive model of Equation 1 (with *c* = 0, since $\tilde{X}i,t$ is centered), that is,

(7) $$\begin{array}{cc} \tilde{X}i,t & =\phi i{\tilde{X}}_{i,t-1}+\epsilon i,t\\ \epsilon i,t & ∼N(0,\sigma_{\epsilon }^{2}i). \end{array}$$

At the between-person level (level 2), individual differences in the means *μ_i_*, the autoregressive parameters ϕi, and the residual variances $\sigma_{\epsilon }^{2}i$ are modeled. Typically, it is assumed that *μ_i_*, ϕi, and $\text{log}(\sigma_{\epsilon }^{2}i)$ (i.e., the natural logarithm of the residual variance), come from a multivariate normal distribution (Asparouhov et al., 2018). This implies that the individual mean, inertia, and residual variance can be correlated with each other. Furthermore, the multilevel framework allows us to model these parameters in tandem with other variables. That is, they can be predicted from person characteristics, such as personality traits, psychological well-being, sex, or age, and they can be used to predict later outcomes, such as future health or happiness (for a comprehensive overview, see Koval et al., 2021).

### Normality assumptions

The multilevel AR(1) model as presented above is based on several assumptions (Epskamp et al., 2018; Hamaker et al., 2018), two of which are of particular interest to us here: (a) the residuals at level 1 are normally distributed, and as a result, the within-person fluctuations of *X_t_* are characterized by the normal distribution; and (b) the random effects, including level-2 means, are assumed to come from a multivariate normal distribution. However, these assumptions are not always met in practice (Haslbeck et al., 2023). The broader literature on linear (multilevel) models suggest that regression models are mostly robust against the violation of normality at level 1 (for an overview and discussion, see Knief & Forstmeier, 2021). Furthermore, it has been shown that the violation of the level-2 normality may bias fixed-effect estimates (McCulloch & Neuhaus, 2011), reduce the estimation efficiency and accuracy (Agresti et al., 2004; Schielzeth et al., 2020), and if there is skewness at level 2, make the standard error estimates particularly unreliable (Maas & Hox, 2004). However, to our knowledge, the consequences of such violations in multivariate time series models have not been systematically studied.

**Figure 2.**
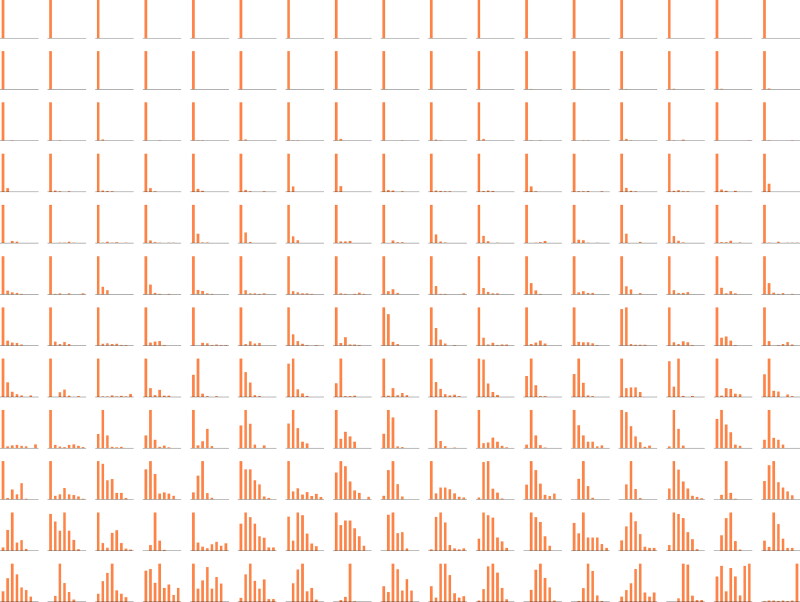
Individual histograms of distress scores (Xi,t) of individuals in the COGITO dataset, sorted by individual means (*μ_i_*).

**Figure 3.**
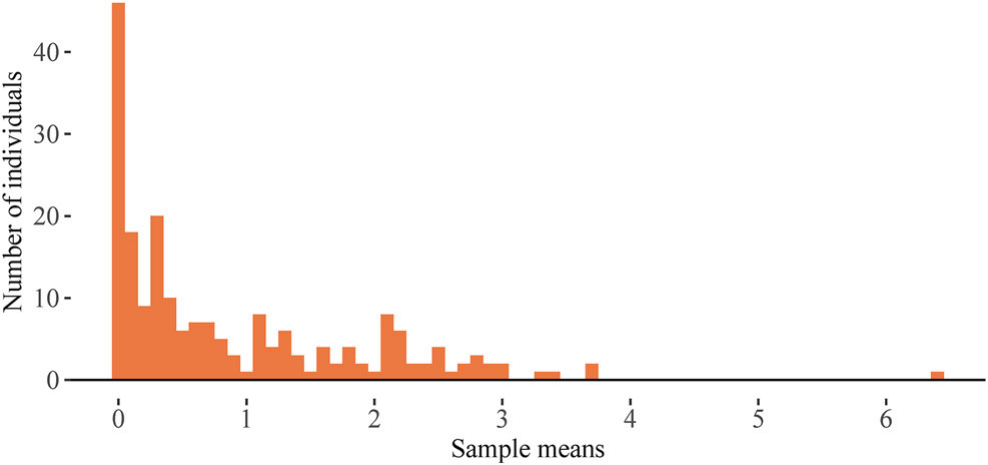
Distribution of sample means of distress scores of 204 individual in the COGITO dataset.

To illustrate the violation of normality assumptions in empirical data, we make use of the intensive longitudinal dataset collected in the COGITO study (Schmiedek et al., 2010), in which 204 adults were measured once a day on various affective and cognitive items for up to 109 days. We focus on the variable *distress* which was measured on a 0–7 Likert scale, resulting in discrete scores. To verify the above assumptions, we look at the individual histograms of Xi,t, and the histogram of *μ_i_*. Figure 2 shows individual histograms of all participants, ordered by individual means. It shows that the distributions of responses gradually become more symmetric as the mean increases. As evident from these plots, the distress score of most of the individuals are remarkably skewed, and furthermore, almost two-thirds of them exhibit a strong floor effect in their scores. This, together with the discreteness of the scores, is a clear indication of the violation of the first assumption of the multilevel AR(1) model. To check the level-2 normality of *μ_i_*, we show the distribution of the sample means in Figure 3. The skewed distribution of person means indicates a violation of the second assumption of the multilevel AR(1). These two assumptions can also be more efficiently verified using summary statistics of the data, as detailed in Appendix A.

**Figure A1.**
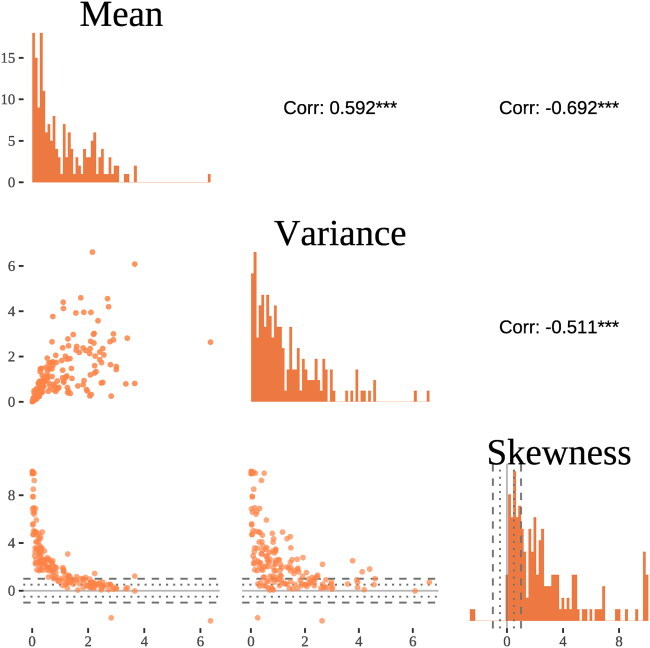
Histograms and pair-wise scatter plots of the individual summary statistics ($\mu_{i},\sigma_{i}^{2},\gamma_{i}$) of distress scores in the COGITO dataset, and the Pearson correlations between them (with *, **, and *** respectively denoting *p* < 0.05, *p* < 0.01, and *p* < 0.001). The dotted and dashed lines, respectively, mark the conventional thresholds of moderate (γ=±0.5) and high (γ=±1) skewness.

Despite the above violations, we may analyze the data using a multilevel AR(1) model and investigate the relationship between the individual differences in the autoregressive parameter and the mean. To this end, we made use of M*plus* version 8.6 (Muthén & Muthén, 2017). Given that the individuals have different degrees of variability (see the middle panel of Figure A1), we included random residual variance in our model. We found a positive association between the random mean and the random autoregressive parameter at level 2 (Corr(μ,ϕ)=0.551, 95% credible interval (CI) [0.415,0.659]). We also analyzed the data using a model with fixed residual variance (a common practice in the psychological literature), which resulted in a larger positive correlation estimate (Corr(μ,ϕ)=0.636, 95% CI [0.517,0.728]). These results are in agreement with other findings, in that individuals with more severe negative conditions (here: higher average distress) tend to be characterized by higher autoregression in their distress scores. However, based on the findings of Terluin et al. (2016), we may be concerned that this positive correlation between the mean and the autoregression might have actually been (partly) due to the floor effect, which is clearly present in the dataset.

## Alternative data-generating models

To be able to study whether individual differences in the floor effect can lead to an inflated correlation between mean and inertia, we need to simulate multilevel data that are characterized by the features discussed in the previous section. Hence, the data from plausible alternative data-generating models (DGMs) should have: a) an autocorrelation function akin to an AR(1) process at the within-person level; b) individual differences in skewness (and variability), which depends on the person-specific mean (i.e., lower mean has more skewness and less variability), but not on the person-specific autoregression; and c) person-specific means that can come from a normal or a skewed distribution at level 2. Additionally, it is important that the DGM can mimic the measurement scales that are typically used in psychological self-report data: Oftentimes such measurements are based on using a Likert scale with a limited number of ordinal scale points (Likert, 1932), or with a (practically) continuous scale, like a 0–100 visually assisted scale (VAS). Finally, we believe it is important to consider DGMs whose parameters and behavior can be explained from a substantive perspective, as this contributes to their credibility as plausible alternatives.

In this section, we present three parsimonious alternative DGMs that meet the above criteria. The reason to consider multiple DGMs is that this allows us, later on, to determine whether particular results in the simulation study are generic to these kinds of skewed data (i.e., shared by all three DGMs), or that certain results are specific to a particular DGM. All of these models, as we will see, can approximate the AR(1) model when specific parameter values are chosen, such that they may produce Gaussian-looking marginal distributions—which motivates using them for skewed and non-skewed time series alike. Below we present the alternative DGMs, which are: a) a generalized AR(1) model with *χ*^2^ residuals (Tiku et al., 1999), which is suitable for generating skewed continuous-valued time series; b) the binomial AR(1) model (McKenzie, 1985), that can generate bounded time series of counts with the floor effect; and c) the discrete AR(1) model (Jacobs & Lewis, 1978) that treats the discrete observations as states, and can produce data with any discrete marginal distribution. To ease comparison, the properties of these DGMs are summarized in Table 1.

**Table 1.** Comparing data-generating models.

	Model			
Properties	AR(1)	*χ*^2^AR(1)	BinAR(1)	PoDAR(1)
Data type	Continuous	Continuous	Bounded discrete	Discrete
Formula	Xt=c+ϕXt−1+ϵt $\epsilon t∼N(0,\sigma_{\epsilon }^{2})$	Xt=ϕXt−1+at $at∼\chi^{2}(\nu )$	Xt=St+Rt St∼Binom(Xt−1,α) Rt∼Binom(k−Xt−1,β)	Xt=PtXt−1+(1−Pt)Zt Zt∼Poisson(λ) Pt∼Binom(1,τ)
Parameters	$c,\phi ,\sigma_{\epsilon }^{2}$	ϕ,ν	*α*, *β*, $\theta =\frac{\beta }{1-(\alpha -\beta )}$	τ,λ
Marginal distribution	$N(\frac{c}{1-\phi },\frac{\sigma^{2}}{1-\phi^{2}})$	No closed form	Binom(k,θ)	Poisson(λ)
Marginal mean	$\frac{c}{1-\phi }$	$\frac{\nu }{1-\phi }$	$k\theta =\frac{k\beta }{1-(\alpha -\beta )}$	*λ*
Marginal variance	$\frac{\sigma^{2}}{1-\phi^{2}}$	$\frac{2\nu }{1-\phi^{2}}$	$k\theta (1-\theta )=\frac{k(1-\alpha )\beta }{{\left[1-(\alpha -\beta )\right]}^{2}}$	*λ*
Marginal Skewness	0	$\frac{2{\left(1-\phi^{2}\right)}^{3/2}}{\sqrt{\nu /2}(1-\phi^{3})}$	$\frac{1-2\theta }{\sqrt{k\theta (1-\theta )}}$	$\sqrt{1/\lambda }$
Conditional expectation	c+ϕXt−1	ν+ϕXt−1	βk+(α−β)Xt−1	(1−τ)λ+τXt−1
ACF (l≥0)	$\rho (l)=\phi^{l}$	$\rho (l)=\phi^{l}$	$\rho (l)={\left(\alpha -\beta \right)}^{l}$	$\rho (l)=\tau^{l}$
Markov	No	No	Yes (Equation S29)	Yes (Equations S38, S39)

### The generalized linear AR(1) model with χ^2^ residuals

As we described in the previous section, the marginal distribution of the AR(1) process (Equation 4) is determined by the distribution of its innovations. Thus, the simplest modification to the Gaussian AR(1) model that may yield a non-Gaussian marginal distribution is to replace its Gaussian innovations with ones from another distribution (Akkaya & Tiku, 2001; Tiku et al., 1999). To create data which can exhibit the floor effect, we would pick a distribution which is strictly non-negative (i.e., has a lower bound of zero) and which can be more or less skewed depending on the parameters chosen, for example, the *χ*^2^ distribution or the Gamma distribution (Lloyd & Warren, 1982; Mathai, 1982a, 1982b). Such asymmetrical innovation distributions have been used to model, for instance, the input, capacity and outflow or reservoirs in hydrology (Phatarfod, 1989; Warren, 1986), or in grain storage problems (Prabhu, 1965). In these systems, the external random input to the system is strictly positive (e.g., more water enters a reservoir or more grain is added to a silo), meaning that a mean-zero Gaussian distribution—which can have negative and positive contributions—would be inappropriate.

Some psychological researchers have suggested that these types of models may be appropriate for modeling variations in processes such as negative affect or distress, based on a reservoir analogy (Bergeman & Deboeck, 2014; Deboeck & Bergeman, 2013). Following Deboeck and Bergeman (2013), let us assume *X_t_* represents a person’s level of distress at time *t*, defined as a continuous variable. As the person goes about their daily life, stressful life events occur and contribute to the person’s distress. This addition can be modeled by a random term *a_t_*. Because the events can only add to the person’s distress, *a_t_* should follow a distribution with strictly non-negative values (Aksoy, 2000; Mathai, 1982b), which can be modeled, for example, using the *χ*^2^ distribution with *ν* degrees of freedom (Mulder, 2018). Rather than building up indefinitely, the individual attempts to regulate their distress by gradually dissipating it over time. Their ability to regulate distress away is determined by the parameter ϕ, which controls the dissipation rate; if ϕ is close to zero, then the person is very good at regulating the distress levels, while if it is close to 1, they struggle with it. To take the reservoir analogy, we may imagine the distress process as a water tank which contains a liquid representing distress, and the amount of distress at any time *t* can be measured by the height of the liquid. Stressful events increase distress in the system by randomly adding some liquid to the tank, and the tank has a tap at the bottom which, at each time point, dissipates a proportion (1−ϕ) of the liquid that was in the tank at the previous time.^3^ Put together, the amount of distress in the system (i.e., the height of the liquid) at time *t* is given by

(8) $$\begin{array}{c} Xt=\phi Xt-1+at,at∼\chi^{2}(\nu ), \end{array}$$

in which ϕXt−1 is the leftover distress that stayed in the system from the previous time point and *a_t_* is the random added distress due to stressful events.

We call the model in Equation 8 the *χ*^2^AR(1) model. Unlike the normally distributed innovations of the AR(1) model, *a_t_* may not take negative values and can only push the system further away from its mean. Consequently, *a_t_* can no longer be thought of as “random shocks” to the system, and the *χ*^2^AR(1) model implies a new type of dynamics in which only the passage of time can decrease the person’s distress. The conditional expectation of this model, based on its value at the previous time point, is given by

(9) $$\begin{array}{cc} E[Xt|Xt-1] & =E[\phi Xt-1+at|Xt-1]\\ & =\phi E[Xt-1|Xt-1]+E[at|Xt-1]\\ & =\phi Xt-1+E[at]\\ & =\phi Xt-1+\nu , \end{array}$$

which is comparable to Equation 2, in that both are the sum of a constant (*c* or *ν*) with a leftover from the previous time point (ϕXt−1). As proven in the Supplemental Materials, the ACF of the *χ*^2^AR(1) model is identical to that of the AR(1) model, which is $\rho (l)=\phi^{l}$ for lag l≥0.

Since the (infinite) weighted sum of *χ*^2^-distributed random variables lacks an analytical probability density function (see Di Salvo, 2008), the *χ*^2^AR(1) model does not have a closed-form marginal distribution (Tiku et al., 1999). However, we may analytically calculate its mean, variance, and skewness. As shown in the Supplemental Materials, the marginal mean of the *χ*^2^AR(1) process of Equation 8 is given by

(10) $$\begin{array}{c} E[Xt]=\mu \chi^{2}\text{AR}(1)=\frac{\nu }{1-\phi }. \end{array}$$

When comparing this to the marginal expected value of the AR(1) model in Equation 3, it can be seen that both consist of the constant term of the conditional expectation (compare Equations 2 and 9) divided by 1−ϕ (the dissipation rate). The marginal variance of the *χ*^2^AR(1) process is, akin to the AR(1) process, equal to the variance of the stochastic term (here: 2ν) divided by $1-\phi^{2},$ that is

(11) $$\begin{array}{c} \text{Var}[Xt]=\sigma_{\chi^{2}\text{AR}(1)}^{2}=\frac{2\nu }{1-\phi^{2}}. \end{array}$$

Finally, the marginal skewness of the *χ*^2^AR(1) process can be calculated by

(12) $$\begin{array}{c} \text{Skewness}[Xt]=\gamma \chi^{2}\text{AR}(1)=\frac{2{\left(1-\phi^{2}\right)}^{3/2}}{\sqrt{\nu /2}(1-\phi^{3})}. \end{array}$$

**Figure 4.**
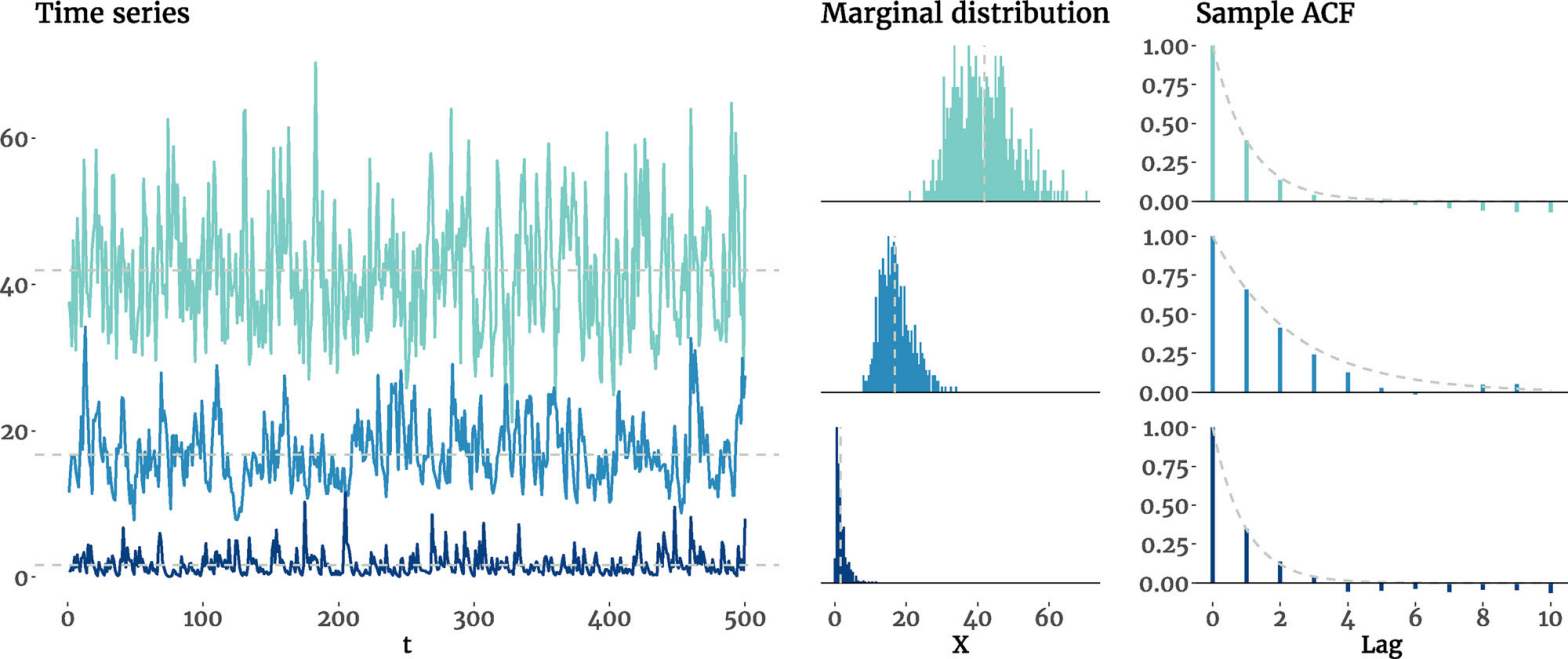
Time series plots, marginal distributions, and the sample ACF of three simulated *χ*^2^AR(1) processes with different degrees of freedom ($\nu^{2}$) and autoregressive parameters (ϕ). In the first process, we have *ν* = 25 and ϕ=0.4, which produce a distribution with $\mu =41.72,\sigma^{2}=58.53,$ and γ=0.48; the second process has *ν* = 5 and ϕ=0.7, resulting in $\mu =16.68,\sigma^{2}=19.31,$ and γ=0.62; and the last process has *ν* = 1 and ϕ=0.4, leading to $\mu =1.67,\sigma^{2}=2.32,$ and γ=2.26. The dashed lines in the first two columns mark the mean, and in the right column trace the exponential decay of the theoretical ACF.

Thus, the conditional expectation, ACF, and the marginal mean and variance of the *χ*^2^AR(1) model parallel those of the AR(1) model. It can further be shown that, for large enough values of *ν*, the *χ*^2^ distribution can be approximated by a Gaussian distribution with N(ν,2ν) (O’Neill, 2019), making the input term *a_t_* of Equation 8 similar in shape to the innovation term *ϵ_t_* of the AR(1) model. In Figure 4 we show three simulated *χ*^2^AR(1) processes with different parameters and their marginal distributions and sample ACFs. As evident in the plots, increasing *ν* makes the marginal distribution more symmetrical (which was expected, as *ν* appears in the denominator of skewness in Equation 12), making it more Gaussian-like (see the Supplemental Materials). Furthermore, because *ν* and ϕ can be set independently, it is possible to have processes with the same autoregressive parameter, while they differ in all their distributional properties.

### The binomial AR(1) model for bounded count time series

In many contexts, we are faced with discrete-valued time series that often concern the *counts* of things (Campbell & Walker, 1977; Cardinal et al., 1999; Jung et al., 2006). Here we consider the binomial AR(1) (BinAR(1)), first presented by McKenzie (1985), which can model count time series that have an upper bound and may also be expressed with a parsimonious Markov model (see the Supplemental Materials). To explain its usefulness in the context of clinical psychology, we make use of the following metaphor. Assume we are measuring a person’s distress by asking them to rate it on a Likert scale from 0 (not at all) to *k* (very much). When using the BinAR(1) model, this implies that we interpret such a scale as representing the number of units of distress that the person feels, and that they have an emotional capacity of feeling a maximum of *k* units of distress. We can think of each unit being represented by a light bulb that can be either on or off; the person’s level of distress is then the brightness of their emotion, which is determined by the number of distress bulbs that are switched on. These light bulbs do not have any particular order, and hence only the number of light bulbs switched on is of interest. When the participant rates their distress on the Likert scale, this comes down to them “counting” how many of those bulbs are turned on, which is then represented by the score *X_t_*. The temporal series of such measurements would therefore comprise a time series of counts.

In the BinAR(1) model the number of lit light bulbs *X_t_* is expressed as a function of the number of light bulbs that were *switched on* at the previous occasion (i.e., *X_t_*_−1_), and the number of light bulbs that were *switched off* at the previous occasion (i.e., k−Xt−1). Specifically, *X_t_* can be expressed as the sum of two binomially distributed variables, that is

(13) $$\begin{array}{c} Xt=St+Rt,\text{ where }\{\begin{array}{c} St∼\text{Binom}(Xt-1,\alpha )\\ Rt∼\text{Binom}(k-Xt-1,\beta ) \end{array}. \end{array}$$

**Figure 5.**
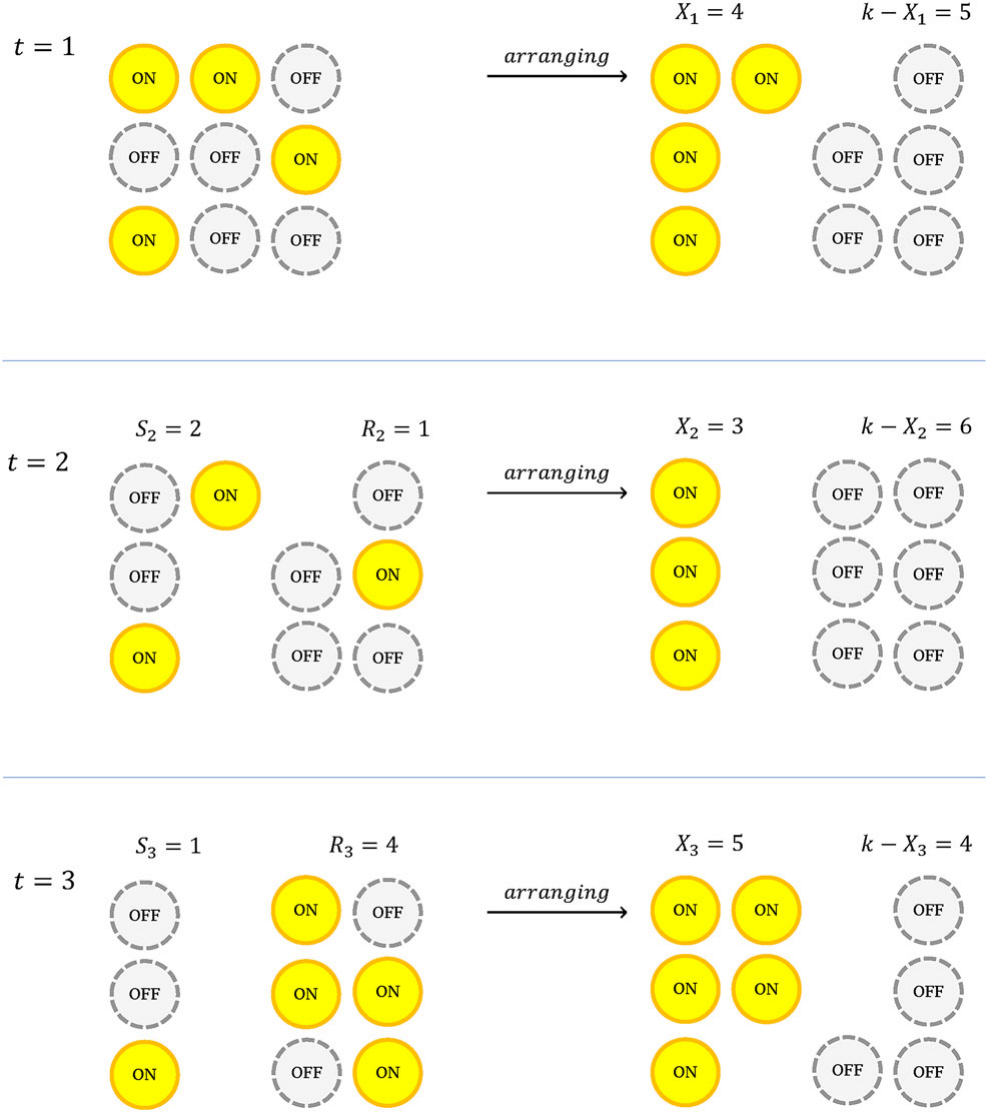
An example of a BinAR(1) process with *k* = 9 for three measurement occasions. At each time point *t*, a number of light bulbs are turned on (*X_t_*), and the rest (i.e., k−Xt) are switched off. The number of lit light bulbs at time *t* depends on two sets of light bulbs; those that were turned on at *t* – 1 and remained lit at *t* (*S_t_*, for survivors), and those that were switched off at *t* – 1 but turned on at *t* (*R_t_*, for revived light bulbs). Because the light bulbs are replaceable, at each time, we rearranged them such that it becomes clear that *S_t_* is a subset of *X_t_*_– 1_ and *R_t_* is a subset of k−Xt−1.

The component *S_t_* can be understood as the number of light bulbs that were turned on at the previous occasion *X_t_*_−1_, and that are still on at occasion *t* (i.e., the “survivors”), with *α* being the probability of a light bulb to remain on (i.e., the *survival probability*). The component *R_t_* is the number of light bulbs that were off at the previous occasion (i.e, k−Xt−1), but are switched on at occasion *t* (i.e., the “revived” bulbs), with *β* being the probability of switched-off light bulbs to be turned on (i.e., the *revival probability*). Figure 5 shows an illustration of the light bulb metaphor for a BinAR(1) process with *k* = 9.

Since the number of light bulbs that are on at occasion *t* (i.e., *X_t_*) depends on the number of light bulbs that were on at the previous occasion *t* − 1 (i.e., *X_t_*_–1_), this process must be characterized by autocorrelation over time. To see how the autocorrelation relates to the probabilities *α* and *β* of the two binomial distributions in Equation 13, we derive the conditional expectation of E[Xt|Xt−1], that is,

(14) $$\begin{array}{cc} E[Xt|Xt-1] & =E[St+Rt|Xt-1]\\ & =E[St|Xt-1]+E[Rt|Xt-1]\\ & =\alpha Xt-1+\beta (k-Xt-1)\\ & =\beta k+(\alpha -\beta )Xt-1. \end{array}$$

The latter expressions shows great similarity to the conditional expectation of the AR(1) model of Equation 2: It contains a constant term (*βk*) comparable to the intercept *c* of the AR(1) model, and an autoregression term (α−β) comparable to the autoregressive parameter ϕ of the AR(1) model. It can be shown that the autocorrelation function of the BinAR(1) model is similar to that of an AR(1) model, given by $\rho (l)={\left(\alpha -\beta \right)}^{l}$ for l≥0 (see the Supplemental Materials for derivations), which confirms the correspondence between the BinAR(1) model’s α−β and the regression coefficient ϕ in the AR(1) model. The 0−k integer values of the BinAR(1) process can be thought of as *k* + 1 distinct *states*, and the BinAR(1) model can also be expressed as a special case of a first-order Markov model that is fully characterized by model’s parameters, *α* and *β*: See the Supplemental Materials for details.

The marginal distribution of the BinAR(1) model follows a binomial distribution (cf. McKenzie, 1985; Weiß & Kim, 2013) with

(15) $$\begin{array}{c} Xt∼\text{Binom}(k,\theta ),\text{ where }\theta =\frac{\beta }{1-(\alpha -\beta )}. \end{array}$$

Given the binomial nature of *X_t_*, the marginal mean of the BinAR(1) model is

(16) $$\begin{array}{cc} E[Xt] & =\mu \text{BinAR}(1)\\ & =k\theta \\ & =\frac{k\beta }{1-(\alpha -\beta )}, \end{array}$$

which is akin to the mean of the AR(1) model in Equation 3, as it is based on dividing the constant term of the conditional expectation by 1−ϕ. The marginal variance of the BinAR(1) model is given by

(17) $$\begin{array}{cc} \text{Var}[Xt] & =\sigma_{\text{BinAR}(1)}^{2}\\ & =k\theta (1-\theta )\\ & =\frac{k(1-\alpha )\beta }{{\left[1-(\alpha -\beta )\right]}^{2}}, \end{array}$$

Finally, the marginal skewness is given by:

(18) $$\begin{array}{cc} \text{Skewness}[Xt] & =\gamma \text{BinAR}(1)\\ & =\frac{1-2\theta }{\sqrt{k\theta (1-\theta )}}. \end{array}$$

**Figure 6.**
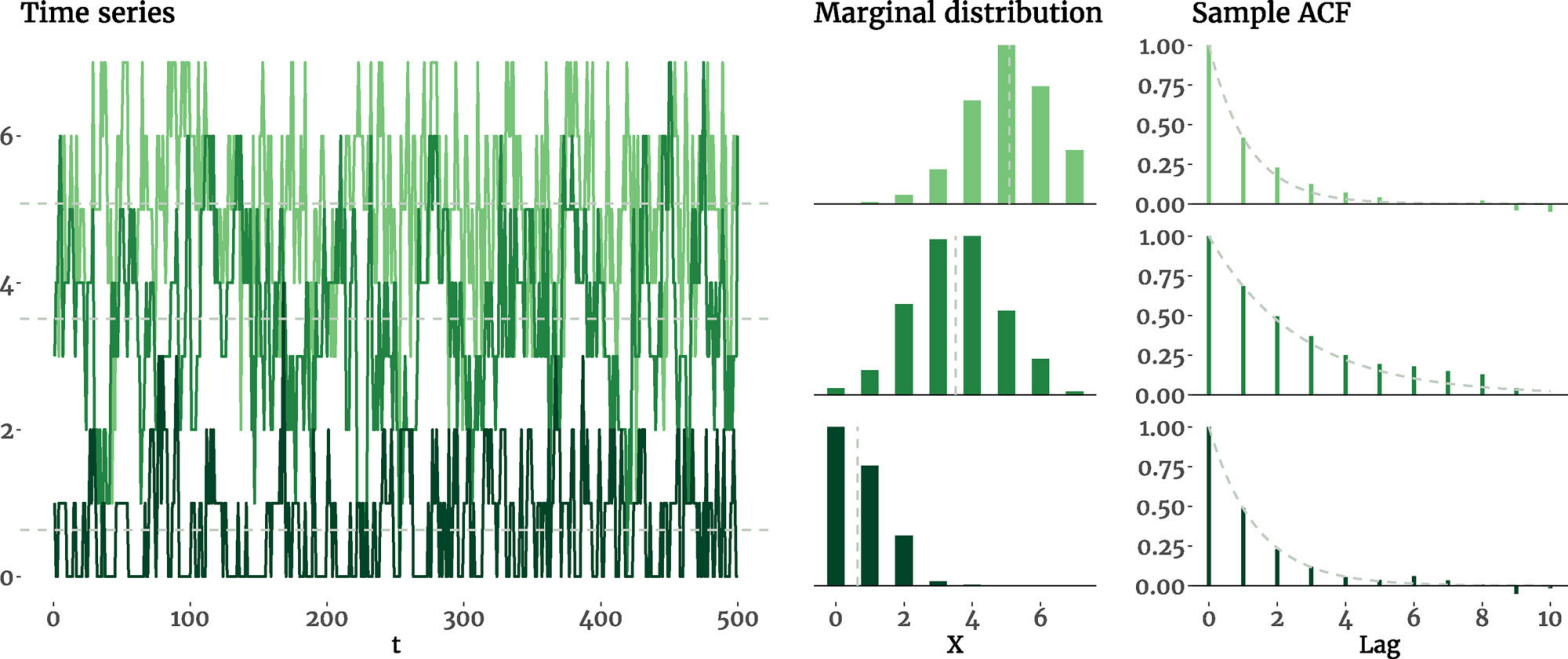
Time series plots, marginal distributions, and the sample ACF of three simulated BinAR(1) processes on a 0−7 Likert scale (*k* = 7) with different survival (*α*) and revival (*β*) probabilities. In the first process, we have α=0.85 and β=0.4, which produce a time series with $\phi =0.45,\mu =5.06,\sigma^{2}=1.37,$ and γ=−0.40; the second process has α=0.85 and β=0.15 resulting in $\phi =0.70,\mu =3.51,\sigma^{2}=1.78,$ and *γ* = 0; and the last process has α=0.5 and β=0.05 leading to $\phi =0.45,\mu =0.65,\sigma^{2}=0.59,$ and γ=1.09. The dashed lines in the first two columns mark the mean, and in the right column trace the exponential decay of the theoretical ACF.

It can be seen that the marginal properties of the BinAR(1) model (Equations 15–18) can all be specified using a single parameter (*θ*). Note that if we only wish to specify a positive autoregressive parameter (ϕ=α−β) without concern for the *α* and *β* values themselves, then we can choose *α* and *β* values for a fixed ϕ that generate any desired value for *θ* (see the Supplemental Materials for details). In Figure 6 we show three simulated BinAR(1) processes with different parameters and their marginal distributions and sample ACFs, which also demonstrate such independence; for instance, although the first and the third processes have different marginal distributions, they share the same autoregressive parameter. Like the *χ*^2^AR(1) model, the BinAR(1) model can approximate the AR(1) model by generating Gaussian-like marginal distributions. Specifically, the binomial distribution with Binom(k,θ) may be approximated by a Gaussian distribution with N(kθ,kθ[1−θ]) (Bagui & Mehra, 2017), and the approximation improves as *k* increases (say, for *k* > 20; Box et al., 1978). Given that the denominator of Equation 18 contains *k*, the skewness of the BinAR(1) model, regardless of *θ*—which is determined by *α* and *β*—approaches zero for larger values of *k*, leading to a symmetrical marginal distribution.

### The discrete AR(1) model for ordinal time series

The second discrete-valued times series model we consider is the discrete AR(1) (DAR(1)) model, which was first presented by Jacobs and Lewis (1978) to model autocorrelated interval or count variables. In contrast to the BinAR(1) model (which was limited to a binomial marginal distribution), the DAR(1) model can be used for any type of count time series with any desired marginal distribution. Later extensions of this model include versions that can handle, for instance, ordinal (e.g., Biswas & Song, 2009; Pegram, 1980; Weiß, 2020) or categorical variables (see, e.g., Biswas et al., 2014; Weiß, 2020). Here, we focus on the original DAR(1) model, which is suitable for integer-valued time series. We start with presenting the DAR(1) model in its general form, and demonstrate this model via an illustrative example of modeling distress using a version of the DAR(1) model with the Poisson marginal distribution, which is tailored for modeling counts of stressful events occurring with a constant rate.

Assume that, in a daily diary study, we measure the daily distress of a person. To use the DAR(1) model, we should assume that distress can be modeled by a collection of small, independent *distress units*, meaning that the person’s level of distress at any time would be equal to the *counts* of such units that the person is experiencing. We ask the person to report their distress on a discrete scale of non-negative integers, which need not have an upper bound. At the start of the experiment, the person starts with a distress level equal to some integer value of *X*_1_. Based on Frijda’s “law of conservation of emotional momentum,” which posits a resistance of one’s mental states to change when nothing happens (Frijda, 1988, 1992), we may assume that the individual remains at the same level of distress from one measurement occasion to the next, unless some kind of influence acts on their distress levels. The influence is independent of the stress levels that the person is exposed to, and may be due to an external event (like getting a call from a close friend or hearing sad news), or an internal one (like a new thought about a friend’s wedding, a traumatic memory that comes up, or some hormonal changes). In the DAR(1) model, whether or not such an influential event takes place can be modeled using a binary variable *P_t_*, which models the *persistence* of the emotion. When such an event is absent on day *t* = 2 (i.e., the emotion *persists*, P2=1), the person will keep the same level of distress as yesterday (*X*_2_ = *X*_1_); in contrast, if such an event took place (i.e., P2=0), the person’s distress level will be equal to the amount of stress to which the person is exposed today. We assume that the amount of external stress at any time *t*, denoted by *Z_t_*, can also be modeled by a number of independent *stress units*; thus, if P2=0, the person’s level of distress will be *X*_2_ = *Z*_2_.

By extending this process to other days, the DAR(1) model for the person’s daily distress can be expressed as

(19) $$Xt=\{\begin{array}{cc} Zt, & \text{if}Pt=0\\ Xt-1, & \text{if}Pt=1 \end{array}.$$

Since the influential events are independent from each other and independent from *Z_t_*, we may model *P_t_* with a Bernoulli process (or a binomial process with *k* = 1), in which the probability of an emotion persisting (and so, the probability of no event occurring to impact the time series) is equal to *τ*, meaning that Pt∼Binom(1,τ).

With the model for persistence in place, we must now specify a model for the external stressful events, *Z_t_*. We assume that the number of stress units over time are independent from each other and follow a discrete distribution Π, that is, Zt∼Π. Because *X_t_*, the person’s felt distress today, is equal to either yesterday’s felt distress (*X_t_*_−1_) or today’s number of external stress units (*Z_t_*), and given that *Z_t_* of today is independent of *X_t_*_– 1_ of yesterday, we may conclude that *X_t_* and *Z_t_* are independent from each other. Despite this independence, as shown in the Supplemental Materials, the marginal distribution of person’s distress (*X_t_*) follows the same distribution as *Z_t_*, meaning that Xt∼Π. Note that the independences of *Z_t_*, *P_t_*, and *X_t_* entail that the person, in case an impact takes place (*P_t_* = 0) may or may not experience the same level of distress as yesterday. In total, the person’s felt distress is fully characterized by two independent processes: *Z_t_*, that captures the tendency of being exposed to different number of stress units; and *P_t_*, the person’s tendency of not being impacted by influential events. Consequently, individual differences in felt distress is characterized by individual differences in the distribution of *Z_t_* (i.e., Π) and the probability of persistence (i.e., *τ*).

To infer the properties of the DAR(1) process, we rewrite Equation 19 as

(20) Xt=PtXt−1+(1−Pt)Zt.

Given the said independences, we may find an expression for the conditional expectation of *X_t_* for the DAR(1) model, that is

(21) $$\begin{array}{cc} E[Xt|Xt-1] & =E[PtXt-1+(1-Pt)Zt|Xt-1]\\ & =E[PtXt-1|Xt-1]+E[(1-P_{t})Zt|Xt-1]\\ & =E[Pt|Xt-1]E[Xt-1|Xt-1]+\text{Cov}(Pt,Xt-1|Xt-1)\\ & +E[1-Pt|Xt-1]E[Zt|Xt-1]+\text{Cov}(1-Pt,Zt|Xt-1)\\ & =E[Pt]Xt-1+0+E[1-Pt]E[Zt]+0\\ & =\tau Xt-1+(1-\tau )E[Zt]. \end{array}$$

This is similar to the conditional expectation of an AR(1) process (Equation 2) with an intercept c=(1−τ)E[Zt] and a regression coefficient of ϕ=τ.

As shown in the Supplemental Materials, the autocorrelation function of the DAR(1) model is similar to that of the AR(1) model, and is given by $\rho (l)=\tau^{l}$ for l≥0 (Jacobs & Lewis, 1978). Note that, as *τ* is a probability between 0 and 1, unlike the AR(1) model, the DAR(1) model cannot account for negative autoregressive parameters.

The DAR(1) model, as we formulated above, does not impose any restrictions on the distribution of *Z_t_* as long as it is integer-valued; Π may be bounded, like the binomial distribution (alluding to the light bulb metaphor of the BinAR(1) model) or the beta-binomial distribution, or unbounded, like the Poisson distribution or the negative binomial distribution. Here we assume that *Z_t_* follows a Poisson distribution, that may produce marginal distributions with varying degrees of skewness, and furthermore, it has a substantive appeal based on the metaphor of stress units: If we assume that, on any given day, the person is exposed to, on average, *λ* stress units, *Z_t_* (and consequently, *X_t_*) would follow a Poisson distribution with rate parameter *λ* (i.e., Zt∼Xt∼Poisson(λ)). In this case, the probability of the person being experiencing a distress level equal to *u* is given by the probability mass function of the Poisson distribution, that is,

(22) $$P(Xt=u)=\frac{\lambda^{u}e^{-\lambda }}{u!}.$$

We call this model the Poisson DAR(1) model, or the PoDAR(1) model in short. Based on the properties of the Poisson distribution, the marginal mean, variance, and skewness of the PoDAR(1) model can be calculated given the rate parameter *λ*:

(23) $$\begin{array}{cc} E[Xt] & =\mu \text{PoDAR}(1)=\lambda ,\\ \text{Var}[Xt] & =\sigma_{\text{PoDAR}(1)}^{2}=\lambda ,\text{ and}\\ \text{Skewness}[Xt] & =\gamma \text{PoDAR}(1)=\sqrt{1/\lambda }. \end{array}$$

**Figure 7.**
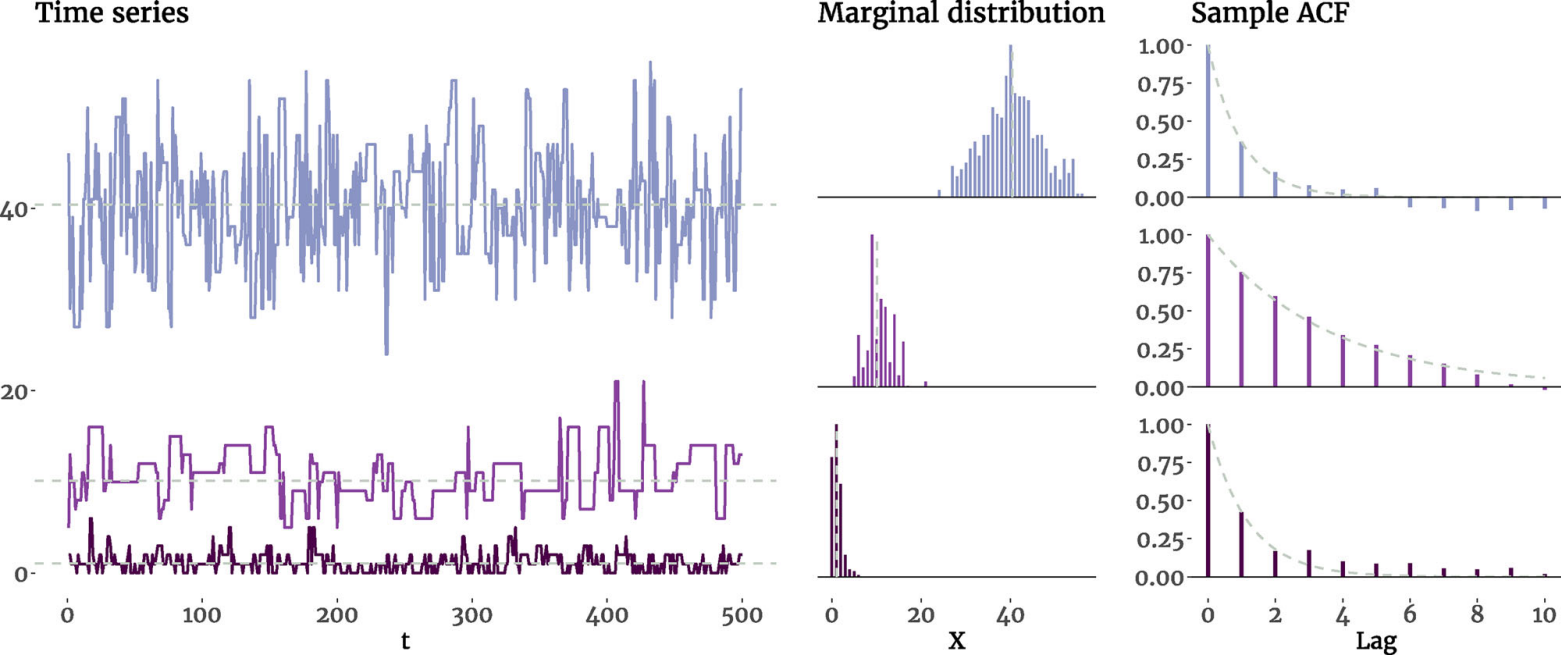
Time series plots, marginal distributions, and the sample ACF of three simulated PoDAR(1) processes with different rate (*λ*) and persistence (*τ*) parameters. In the first process, we have *λ* = 40 and τ=0.4, which produce a time series with $\phi =0.40,\mu =40.17,\sigma^{2}=40.11,$ and γ=0.16; the second process has *λ* = 10 and τ=0.8, resulting in $\phi =0.80,\mu =10.08,\sigma^{2}=9.90,$ and γ=0.35; and the last process has *λ* = 1 and τ=0.4, leading to $\phi =0.40,\mu =0.98,\sigma^{2}=0.99,$ and γ=1.05. The dashed lines in the first two columns mark the mean, and in the right column trace the exponential decay of the theoretical ACF.

Like the previous two models, the PoDAR(1) model can also approximate the AR(1) model and produce Gaussian-like marginal distributions; it is known that a Poisson distribution with rate parameter *λ* may be approximated by a Gaussian distribution with N(λ,λ), especially for larger values of *λ* (Bagui & Mehra, 2017; Govindarajulu, 1965). In that case, given that *λ* appears in the denominator of skewness (Equation 23), for relatively large rates (e.g., λ>10) the skewness becomes negligible (γ<0.32). In Figure 7 we show three simulated PoDAR(1) processes with different parameters and their marginal distributions and sample ACFs. Comparing the first and the third process shows that with the same autoregressive parameter *τ*, different marginal properties can be achieved by different values of *λ*, which confirms the independence of the dynamic and marginal properties of the PoDAR(1) model. Furthermore, it is worth noting that the PoDAR(1) model, like the BinAR(1) model (of Equation 13), can also be expressed as a special case of a first-order Markov process characterized by only two parameters, *τ* and *λ*. See the Supplemental Materials for details.

## Simulation study

It has been argued that the staging effect may be an artifact due to modeling skewed data (Terluin et al., 2016). In the context of time series data and autoregressive models, this may imply that we find lower autoregression for individuals with lower means, simply because their data are more impacted by the floor effect, and thus have lower variability and more skewness. As a result, a positive association (correlation or covariance) between an individual’s mean and their autoregressive parameter may not reflect a meaningful property that requires a substantive interpretation, but could simply be a result of using a model that does not properly account for such distributional differences which characterize the data from different individuals. We focus on skewness as an effective indicator for the strength of the floor effect (see Appendix A), which also directly quantifies the degree of non-normality in time series data.

To investigate whether the failure to account for skewness results in bias in the estimation of the autoregressive parameter in a multilevel AR(1) model, we simulated data where individuals were characterized by different degrees of skewness (violating the first assumption of the multilevel AR(1) model), and studied whether there was an artifactual relationship between the estimated mean and autoregression. Specifically, their skewness was related to their mean, in that individuals with lower means were characterized by more severe skewness, whereas individuals with higher means had more symmetric distributions. We used the various models described before to generate such data: The AR(1) model (which has normally-distributed residuals; Equation 1); the *χ*^2^AR(1) model (Equation 8); the BinAR(1) model (Equation 13); and the PoDAR(1) model (Equations 20 and 22). Furthermore, in each dataset, we randomly sampled the autoregressive parameters of individuals from a normal distribution, independent of the means. This necessarily implies that the autoregressive parameter is not associated with any other distributional feature (i.e., mean, variance, or skewness). Hence, when using a multilevel AR(1) model to estimate a random mean and a random autoregressive parameter, the correct result would be to find a correlation of zero between these two parameters, whereas a positive correlation would form evidence for the hypothesis that staging is an artifact of skewed data.

We took a Bayesian multilevel analysis approach—rather than multiple parallel *N* = 1 analyses—to estimate both within- and between-person dynamics simultaneously. This had two advantages: (a) it allowed us to include random (i.e., person-specific) residual variance to investigate whether this affected the possible bias; and (b) it minimizes Nickell’s bias in the estimation of the autoregressive parameter (Nickell, 1981) that arises when using a frequentist multilevel modeling approach with observed mean centering (Hamaker & Grasman, 2014). In general, we opted for a modeling approach that could be considered common in this area; that is, we assumed all random effects had a normal distribution, even though this may have deviated from our data-generating mechanism. The goal was to investigate whether these typical assumptions would lead to artificial positive correlations. To this end, we examined the right-sided Type-I error rate, that is, the estimated probability of discovering a positive correlation (between the estimated mean and autoregressive parameter) while the true correlation, per our simulation design, was zero. Furthermore, we estimated the bias in the estimated correlation by studying how far off were the point estimates of the correlation from its true value (of zero).

### Generating the datasets

To generate multilevel data according to the various DGMs, we first chose the level-2 parameters, and then simulated the data per person. Throughout, we sampled the model-implied autoregressive parameter for each individual in each DGM from a normal distribution with ϕi∼N(0.4,0.01). By doing so, the assumption of normality for the distribution of the level-2 autoregressive parameter was upheld (Asparouhov et al., 2018). At level 2, we considered two distributions for individual means: a Gaussian distribution, and a *χ*^2^ distribution. The first matched the assumptions in standard multilevel software, but the disadvantage of using a Gaussian distribution for the individual means was that only very few people would get very low means, and as a result, only a few people in the sample would be characterized by serious degrees of skewness in their data. The *χ*^2^ distribution allowed for a larger proportion of individuals with a mean close to zero, such that they were characterized by less variability and more skewness due to a floor effect. Note, however, that this level-2 distribution violated the assumptions of multilevel software. Comparing these results with those estimated in the level-2 normal condition allowed us to investigate the impact of this form of violation.

After sampling the autoregressive parameter of the models (which is equal to the ACF at lag 1, i.e., ρ(1)), together with the independently sampled mean per person, we determined each model’s parameters per person using the expressions in Table 1. Based on these parameters, time series with varying degrees of skewness were generated using the respective DGM, and this was repeated for all other cases. Hence, we had two level-2 distributions (i.e., Gaussian- and *χ*^2^-distributed), combined with four different level-1 data-generating models (i.e., the AR(1), *χ*^2^AR(1), BinAR(1), and PoDAR(1) models), resulting in eight different DGMs. For each DGM we considered three samples sizes at level-2 (i.e., *N* = 25, 50, 100), combined with three sample sizes at level-1 (i.e., *T* = 25, 50, 100), resulting in nine combinations, totaling 8 × 9 = 72 different conditions. For each condition, we created 1000 replications using R Statistical Software (v.4.1.1; R Core Team, 2021). Figures S1, S2, S4, and S5 show person histograms and distributions of summary statistics of sample simulated datasets of each DGM.

### Analyzing the simulated data

We used M*plus* version 8.6 (Muthén & Muthén, 2017) to analyze each of the 72,000 simulated datasets with two different models. First, we estimated a multilevel AR(1) model with random mean and random autoregressive parameter (allowing individual differences in means and autoregressions) and a fixed residual variance, such that all individuals would have the same residual variance, and estimated the level-2 correlation of the random means and autoregressions. This model is commonly used in clinical research (see, e.g., Hamaker et al., 2018). Since the marginal variance of the AR(1) model is determined by the autoregressive parameter and residual variance (Equation 4), given that the marginal variance varied across individuals, individual differences in marginal variance could result in biased autoregressive parameter estimates per person (cf., Jongerling et al., 2015). Consequently, this might lead to bias in the estimated correlation between the estimated mean and the estimated autoregressive parameter. To eliminate this possible source of bias, we analyzed each dataset with a second model that included random residual variance, allowing individuals to have different means, autoregressive parameters, and residual variances. We used the R packages *MplusAutomation*, *snow*, *future*, and *doFuture* to interface M*plus* from R and run codes in parallel (Bengtsson, 2021; Hallquist & Wiley, 2018; Tierney et al., 2021).

### Results

To evaluate whether differences in the floor effect among individuals led to positive associations between the estimated random autoregressive parameter ϕi and the random mean *μ_i_*—a statistical artifact which would be mistaken as evidence for the staging effect—we studied the estimated level-2 correlation between the random mean and random autoregressive parameter. Based on this, we determined the right-sided Type-I error rate (which we call *positive error*, as it was in the positive direction), which is the probability of mistakenly deducing that the correlation is positive, resulting in erroneous evidence in favor of the staging effect. To quantify this, for each of the condition, we counted the number of times that the 95% credibility interval (CI) of the estimated correlation lied above zero (suggesting a positive association), and divided it by the number of converged replications in the same condition.^4^ Although not directly related to the hypothesized staging effect, we also considered the left-sided Type-I error rate (which we call *negative error*, as it is in the negative direction), to estimate the probability of mistakenly inferring a negative, rather than zero, value for the correlation between the means and the autoregressive parameters.

Furthermore, we quantified the strength of the errors by estimating how far off the estimates of the correlation were from its true value (of zero). To do so, we estimated the empirical bias and the empirical root mean squared error (RMSE). The empirical bias was calculated by taking the average point estimate of the correlation across all of the converged replications within the same condition minus the true value. Similarly, we estimated the empirical RMSE by squaring the difference between the estimated correlation and its true value in each replication, and we calculated the square root of their average among the converged replications within each condition.

#### One-sided Type-I error

As the 95% CI was used to determine whether the estimated random effect correlation between *μ_i_* and ϕi was zero, we considered 5% to be the acceptable threshold for the Type-I error rate; a higher estimated error rate would provide evidence for the hypothesis that the random effect correlation was an artifact. Because we were interested in the directional Type-I error rates, we used 2.5% as the acceptable threshold for such errors (and as the sampling distribution was not necessarily symmetrical, we furthermore considered a more lenient threshold of 5% for the positive and negative error rates; see Appendix B for details). Below we first discuss the results in case of normally distributed means, and then for the more realistic case with *χ*^2^-distributed means.

##### Gaussian-distributed means

**Figure 8.**
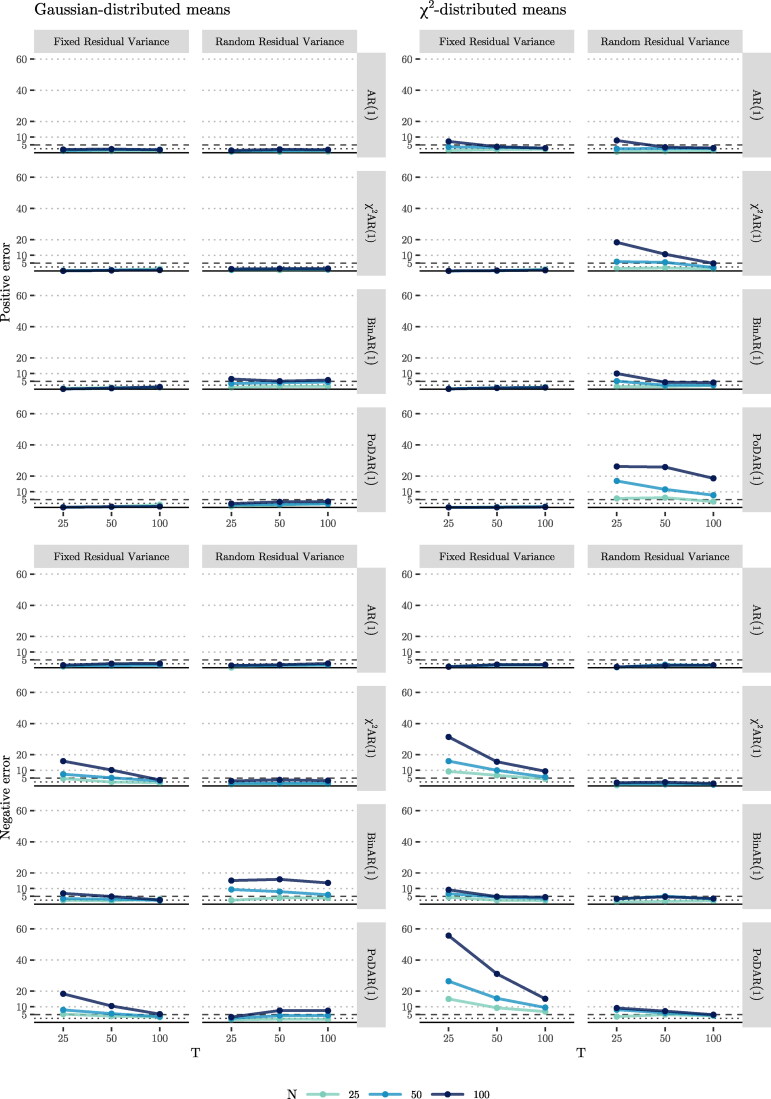
Right-sided (top) and left-sided (bottom) Type-I error rates in the estimated correlation between *μ_i_* and ϕi.

The upper half of Figure 8 includes the positive errors for each condition (i.e., where the correlation is erroneously estimated to be positive). We begin with the model with fixed residual variance on the left. For the AR(1) model, regardless of the sample size *N* and time series length *T*, this rate was below 2.5%, which means that in more than 97.5% of the converged datasets, the 95% CI of the estimated correlations either included zero or was totally below zero. This was expected, as the AR(1) model is identical to the fitted model. For this reason, we regard the results of the AR(1) model as a benchmark, and we assess the results of the other DGMs (also for bias and RMSE) relative to this model. We observe a similar pattern for other DGMs, that is, for all *N* and *T*, the positive error was below 2.5%.

**Figure B1.**
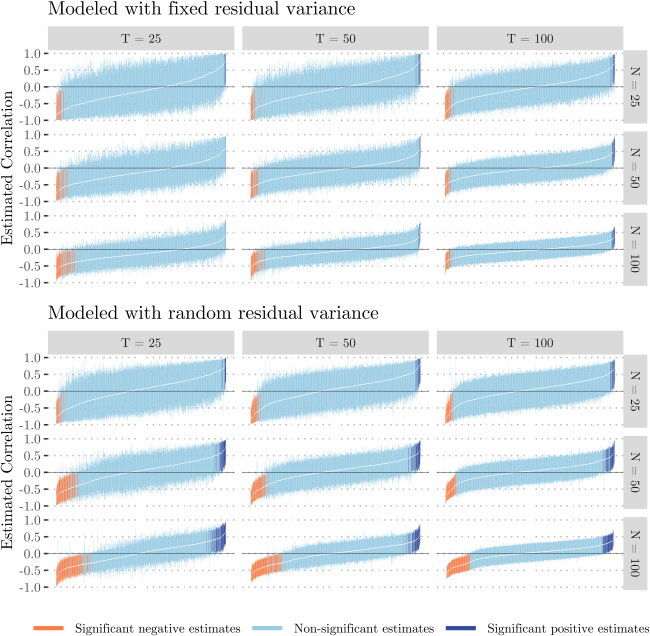
Point estimates (in white) and the corresponding 95% CIs of random effect correlation between *μ_i_* and $\phi_{i}$ for the BinAR(1) model with Gaussian level-2 means. The black line represents the correct value of the correlation (zero). The CIs are ordered based on the point estimates and are colored depending on whether they are below zero (negative Type-I error), overlapping with zero (non-significant estimates), or above zero (positive Type-I error).

By extending the model with random residual variance (see the upper half of the second column of Figure 8), positive errors of the AR(1) and the *χ*^2^AR(1) models remained under 2.5%. In the BinAR(1) model, we observe elevated positive errors for *N* = 50, 100 (getting as high as 4.5 and 6.5%, respectively) which very slightly decreased as *T* increased, but increased as *N* increased. The latter can be explained by the 95% credible intervals of the estimates of the correlations which narrow as the sample size grows, thus making them less likely to cover zero (see Figure B1 in Appendix B). The PoDAR(1) model, in most cases, resulted in positive error rates less than 2.5%, and the increase in *N* elevated positive error for this DGM to around 3.7%.

When considering the negative errors for the model with fixed residual variance (the first column in the lower half of Figure 8), we see that the negative errors of the AR(1) model remained close or under 2.5%. In all other models, the error rates were mostly above 2.5% and reached as high as 15.9, 6.9, and 18.3. In all cases, increasing *T* brought down the error rates, though an increase in *N* noticeably increased them. By using a model with random residual variance (see the second column), the error rate of the AR(1) and the *χ*^2^AR(1) models remained under 3.9%. In the BinAR(1) model, we observe that the negative error, in all conditions, increased noticeably by a factor of up to 3.9 compared to the first column. In the PoDAR(1) model, increasing *T* increased the negative error up to 7.6%. As before, in all cases, an increase in *N* noticeably increased the error rates, though increasing *T* slightly decreased the errors in most cases.

##### χ^2^-distributed means

**Figure B2.**
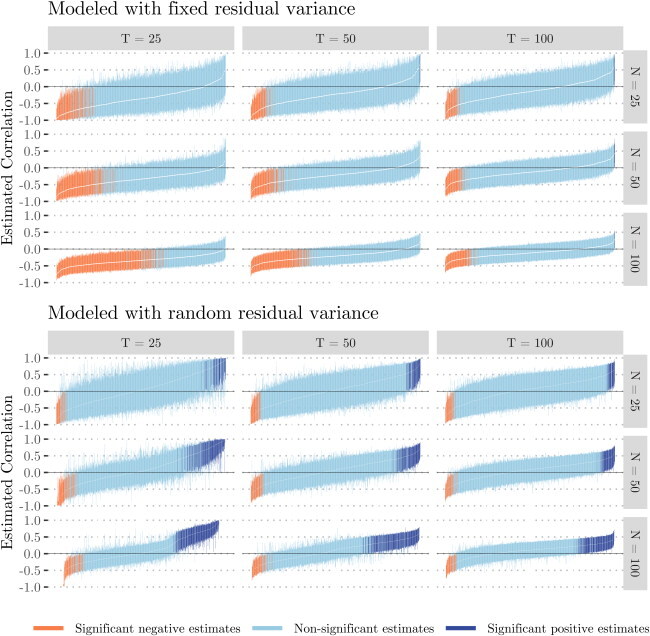
Point estimates (in white) and the corresponding 95% CIs of the random effect correlation between *μ_i_* and $\phi_{i}$ for the PoDAR(1) model with *χ*^2^-distributed level-2 means. The black line represents the correct value of the correlation (zero). The CIs are ordered based on the point estimates and are colored depending on whether they are below zero (negative Type-I error), overlapping with zero (non-significant estimates), or above zero (positive Type-I error).

When considering the positive error rates in the model with fixed residual variance for the cases with *χ*^2^-distributed sample means (see the third column of the upper half of Figure 8), we see that they consistently remained under 2.5% (and very close to zero), with one exception (i.e., the AR(1) model with *T* = 25, 50). By extending the model to include random residual variance (see the fourth column), for the AR(1) model, we observe a similar pattern compared to the third column. For other DGMs, we observe that an increase in *N* remarkably increased the positive errors (which can be attributed to shrunk CIs for larger sample sizes; see, e.g., Figure B2), whereas increasing *T* reduced the positive error in all cases. In these models, the positive error often exceeded the 5% threshold, getting as high as 26.2% in the PoDAR(1) model.

When considering the negative errors for the model with fixed residual variance (the third column in the lower half of Figure 8), we see that the negative errors of the AR(1) model remained under 2.5%, but were above 5% for the other DGMs in most cases, reaching as high as 55.6%. Increasing *T* was associated with a lower error rate while increasing *N* had a strong opposite effect. When we extended the model with random residual variance (see the fourth column), the negative errors shrunk noticeably in all models, and were below 2.5% for the AR(1) and *χ*^2^AR(1) models, and reached up to around 10% in other models. As before, increasing *T* or decreasing *N* reduced the error.

#### Bias and RMSE

##### Gaussian-distributed means

**Figure 9.**
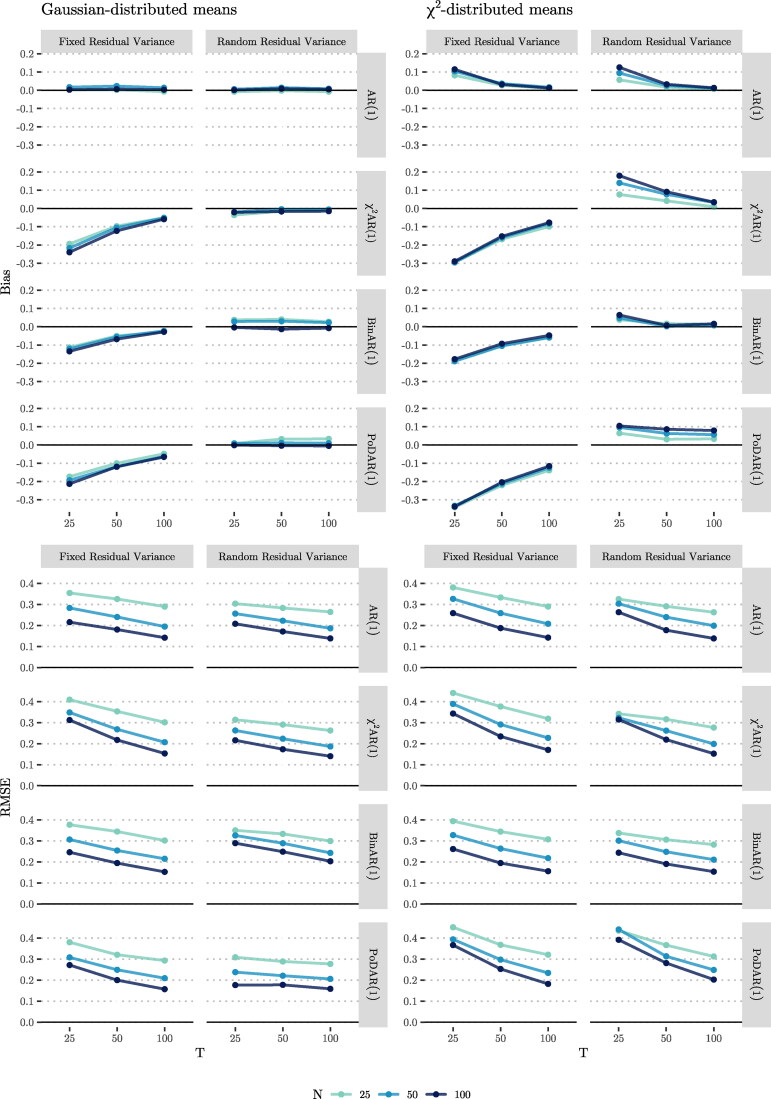
Bias (top) and RMSE (bottom) in the estimated correlation between *μ_i_* and ϕi.

When we consider the bias for the model with fixed residual variance (the first column in the upper half of Figure 9), we see that for the AR(1) model there is no bias in estimating the correlation between the random intercept and the random autoregression, regardless of *T* and *N*. In all other DGMs, we observe bias. However. in contrast to what we had hypothesized based on Terluin et al. (2016), there was a negative rather than a positive bias in the correlation. This negative bias became as large as −0.24. Bias decreased as *T* increased, but *N* had no noticeable effect. When we extended the model to have random residual variance (see the second column), for all alternative DGMs, the bias was considerably smaller than when a fixed residual variance was modeled; in some cases, the bias became slightly positive but remained small (i.e., less than 0.04). In all cases, an increase in *N* reduced the bias, but increasing *T*, overall, had little effect on it.

When considering the RMSE for these scenarios (see the lower left panel of Figure 9), we observe that, in all DGMs, the RMSE of the estimated correlations consistently dropped when either *N* or *T* increased, and the effect of the former was stronger than that of the latter. We observe that, generally, including random residual variance decreased the RMSE somewhat.

##### χ^2^-distributed means

When comparing the results for the model with fixed residual variance when the means followed the *χ*^2^ distribution (see the upper half of the third column of Figure 9), we observe that the bias was up to 60% higher compared to the cases were the means were normally distributed. In the AR(1) model, the bias was equal to 0.114 at its highest (for N=100,T=25). In this model, increasing *T* reduced the bias, while increasing *N* increased the bias, and the effect of the latter was rather small. For the other DGMs, the bias was always negative (reaching −0.342), and increasing *T* (and to a much lesser degree, *N*) brought the bias closer to zero. When we included random residual variance in our model (see the fourth column), the bias in the AR(1) model was comparable to the case analyzed using a model with fixed residual variance. In other DGMs, the bias switched from being negative to being positive, yet smaller in magnitude (ranging from 0.002 to 0.179) in comparison to the model with a fixed residual variance. In these models, an increase in *T* reduced the bias, while an increase in *N* increased bias, which was unexpected. When looking at the RMSEs, we see consistent patterns that were similar to those for Gaussian-distributed means: Increasing either *T* or *N* decreased the RMSE; an increase in *N* had a stronger effect on reducing the RMSE; and including random residual variance in the model reduced the RMSE.

### Conclusion

**Figure 10.**
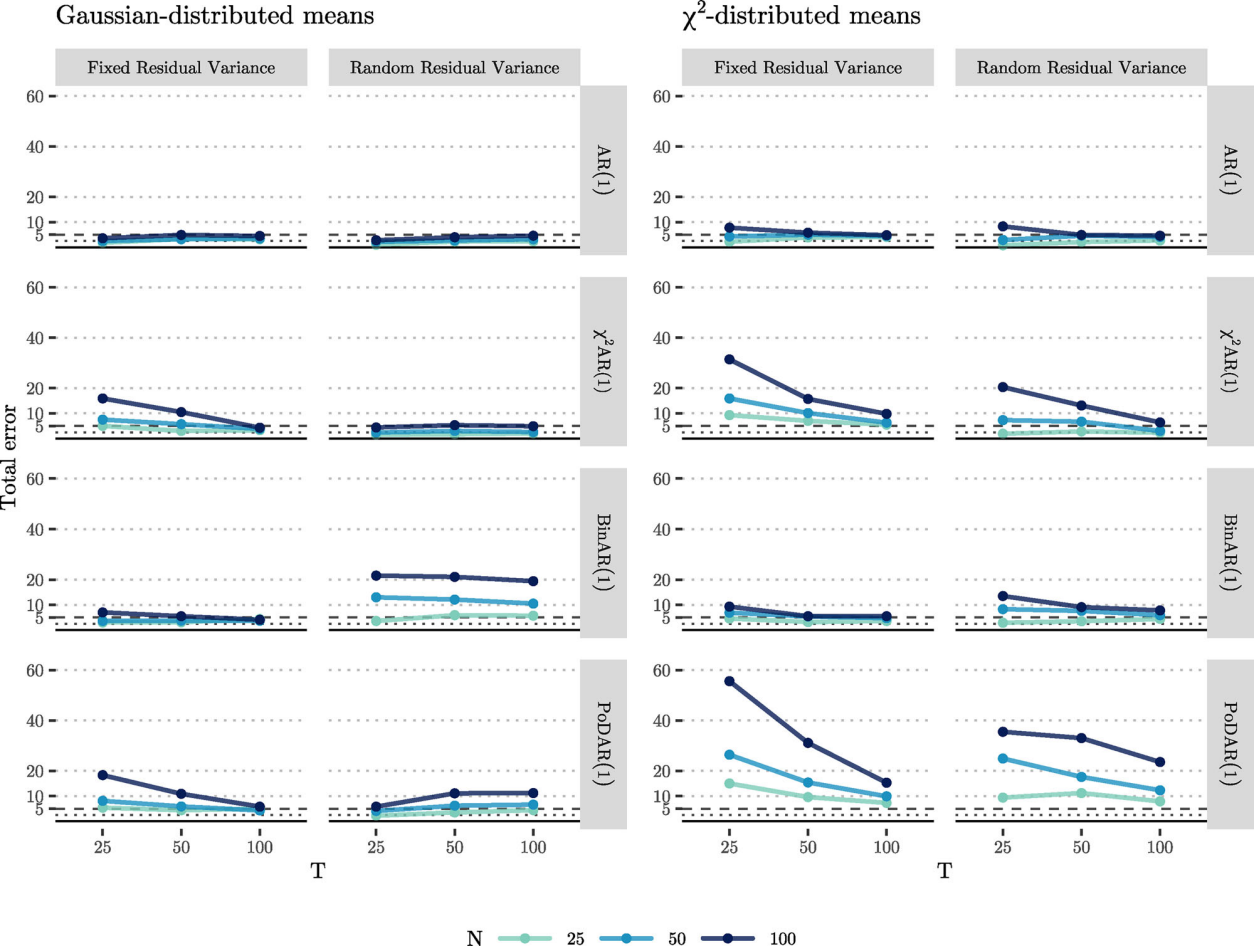
Two-sided Type-I error rate in the estimated correlation between *μ_i_* and ϕi.

In the simulation study described here, we considered multiple DGMs to investigate whether the violation of normality due to skewness at levels 1 and 2 would lead to mistakenly finding a positive relationship between the estimated means and autoregressive parameters in multilevel AR(1) models, when in reality such a relation is absent. Our results provide no evidence for the statistical artifact hypothesis when modeling with fixed residual variance, which is the standard in most empirical applications of multilevel time series models: In all cases we studied, there appeared to be either negligible or a negative, rather than a positive, bias in the estimated correlation between the persons’ means and their autoregressions. Furthermore, the probability of making a positive one-sided Type-I error (i.e., incorrectly concluding there was a positive correlation when the effect was zero) was less than 2.5% in almost all conditions in which the estimated model had fixed residual variance. In contrast though, our results showed that—at least when data were generated with the DGMs considered here—extending the model with random residual variance can lead to positively biased estimates and an elevated probability of making a positive Type-I error. The violation of the normality assumption due to the floor effect always inflated the positive or the negative Type-I error rates, and the two-sided Type-I error rate (that is, the sum of the positive and negative errors) was mostly off the conventionally expected threshold of 5%, reaching up to 55.6% (see Figure 10). We also found that including random residual variance in the multilevel AR(1) model consistently led to decreases in the absolute values of bias and RMSE of the correlation between random effect means and autoregressions, thereby reducing the effect of violating the normality assumptions. Lastly, we observed that an increase in *T* (i.e., longer time series) consistently improved all aspects of estimation, whereas increasing *N* (larger sample size) increased the Type-I error rate and had an inconsistent effect on the bias and RMSE.

Before concluding this section, it is worthwhile to put the magnitude of bias we found in the results into context. In our simulation study, the magnitude of bias in the presence of the floor effect reached up to 0.34, 0.22, and 0.14 in, respectively, short (*T* = 25), moderately long (*T* = 50), and long (*T* = 100) time series, which are considerable when compared with the estimated random effect correlations found in empirical data. For instance, in the distress time series of the COGITO dataset (with around 100 measurements per person), the random effect correlations estimated with the AR(1) model with fixed and random residual variance were, respectively, 0.636 and 0.551; if we can assume that these data were generated by the PoDAR(1) model (where we had the strongest bias), based on our simulation results in the cases with *χ*^2^-distributed means (that are close to the sample means in the distress dataset; see Figure 3) and *T* = 100 (close to the number of measurements per person in the COGITO dataset), we may conclude that the “true”, unbiased random effect correlations estimated by the models with fixed and random residual variance could have been around 0.976 and 0.542.

## Discussion

The autoregressive parameter of the AR(1) model has been used to capture the rigidity or inertia of an emotion or symptom over time, and a rich body of literature has suggested that it is associated with, and can be predictive of, a variety of person characteristics (see Koval et al., 2021). Particularly, it has been suggested that the autoregressive effect in certain affective items is positively correlated with the severity of psychiatric disorders or the mean of the emotion, which has been called the *staging effect* (Wigman et al., 2013). However, some researchers have raised concerns that this observed association may be a statistical artifact of using the multilevel AR(1) model (which assumes the data is Gaussian at level 1 and 2) on time series data that are, especially for negative symptoms of healthier individuals, highly skewed and are characterized by a floor effect (Terluin et al., 2016).

In this paper, we investigated this issue by means of a simulation study. We simulated time series of individuals with with varying degrees of the floor effect and autoregressive parameters that were randomly sampled (independent of the strength of the floor effect), and studied whether analyzing the data with the multilevel AR(1) model could lead to a spurious positive association between the estimated mean and autoregressive parameter at level 2. We considered two versions of the multilevel AR(1) models, with fixed or random residual variance, which have been used in psychological research on inertia. Based on the simulation results, we may conclude that if a model with fixed residual variance is used—a common practice in the psychological literature—the floor effect actually leads to negative, rather than the anticipated positive, bias in the estimated correlation between the random mean and random autoregressive parameter. In contrast, a high right-sided Type-I error rate and a positive bias was found for high *N* and low *T* when a random residual variance was included in the model. However, it should be noted that, to our knowledge, this model type is rarely used in the psychological literature so far, and it is therefore not an explanation for previous empirical findings supporting the staging effect. Notably, this same model, compared to the model with fixed residual variance, has less bias and RMSE in the presence of non-normally distributed level-2 parameters.

While investigating the association between individual means and autoregression has substantive relevance, in order to draw conclusions about what design and analysis choices researchers should make in practice, it is necessary to specify more specific research goals. For instance, if researchers are conducting a confirmatory hypothesis test regarding the presence of a non-zero correlation between the mean and the autoregressive parameter, then the one- or two-sided Type-I error rates are primarily of interest. For example, when the staging effect hypothesis (a positive correlation) is specifically put to test, the researcher should focus more on the positive Type-I error rate, thus a model with fixed residual variance should be favored over a model with random residual variance, and, perhaps counter-intuitively, researchers should favor collecting long time series (high *T*) over larger *N*; when the model is misspecified, we have seen that high *N* and low *T* lead to high Type-I errors due to a combination of bias and too narrow CIs. On the other hand, if the study is more exploratory or descriptive in nature, researchers may care less about hypothesis tests, and care more about the generalizability of their parameter estimates to new samples. In that situation, researchers may care about obtaining estimates which have both low bias and low variability, as indicated by a low RMSE in our simulation study. In that case, researchers might choose to use models with random residual variance, and both sufficiently large *N* and large *T* are important (though researchers should favor higher *N*). Of course, substantive knowledge and beliefs can also play a role in guiding analysis choices, such as expecting participants to have random residual variance, or having reasons to believe that some of the data-generating models we have studied here are more or less plausible than others.

Given that the above decisions have notable consequences and may lead to contrasting conclusions, researchers should be clear about the hypotheses they put to test and communicate them transparently, and if possible, pre-register their studies to prevent hypothesizing after the results are known (HARKing; Kerr, 1998) or other questionable research practices (John et al., 2012; Nosek et al., 2019, Nosek et al., 2018). Finally, given that the strength of the potential bias in the results depends on data characteristics, importantly, the amount of skewness at level 1 and 2, the researchers are advised to investigate—and report—individual histograms and the distributions of means, variances, and skewnesses in the sample (see Appendix A for details). This would help them to have a rough idea about the size of the bias in the results; for instance, if most of the individuals have relatively symmetrical distributions, there is no need to be greatly concerned about over- or underestimating the random effect correlation.

It should be noted, however, that by focusing on different aspects of model fit in isolation from each other, we might fail to see the forest for the trees: In the presence of individuals with skewed response patterns—even if the level-2 normality is not violated—the two-sided Type-I error rates (i.e., the probability of mistakenly detecting a non-zero correlation, when in reality it is zero) is hardly negligible. Furthermore, as discussed in Appendix A, the violation of level-1 normality due to the floor effect often brings about a violation of level-2 normality, which in turn substantially inflates the Type-I error rate (especially for larger sample sizes) and leads to less accurate estimates. One may consider such violations of the assumptions of the multilevel AR(1) model in certain affective time series—and the biases and errors thereof—to be an inevitable consequence of fitting a simple, parsimonious mathematical model to real-world phenomena; nevertheless, the fact that “all models are wrong” does not necessarily undermine their usefulness in abstracting complex phenomena (Box, 1979). On the other hand, the above issues could also be taken to imply that the said affective processes are indeed generated by other *kinds of* mechanisms that have different substantive explanations.

In this paper, we presented three alternative data-generating models with lag-1 serial dependence which can produce marginal distributions that arise in empirical data (i.e., skewed and often times discrete-valued) that the AR(1) model with Gaussian innovations fails to generate. Additionally, their dynamic and distributional properties can be fully specified, independently, using only two parameters—making them more parsimonious than the AR(1) model (which is identified with three parameters). Each of these alternative models is based on different modeling assumptions about the processes underlying empirical data that parallel various substantive interpretations: The *χ*^2^AR(1) model assumes that the external events can only affect the system in one direction (by increasing the levels of the variable) and the decrease in levels is only achieved via a deterministic process (of “dissipation”); the BinAR(1) model assumes that the level of the variable, which is measured as an integer, is determined by the aggregate activity of a set of independent latent units that contribute equally to the level of the variable; and the PoDAR(1) model, alluding to Frijda’s hypothesized law of conservation of emotional momentum (Frijda, 1988), posits that a person has a tendency of experiencing different levels of an affective variable, though the level of this variable only changes under the influence of other (unmeasured or random) factors, which determine the temporal dynamics of the measurements. We did not explore whether these assumptions hold for the underlying data-generating mechanism of specific empirical time series, thus we had no reason to choose one model over the others in our simulation study. Yet, although the results varied across models, they all painted a coherent picture of the effect of the floor effect on the parameter estimates of the multilevel AR(1) model.

These alternative models (and other time series models with non-Gaussian or discrete marginal distributions; see, e.g., Davis et al., 2021; Grunwald et al., 1995; Inouye et al., 2017) not only may lead to more accurate abstractions of affective time series, but also afford the researchers the opportunity of empirically testing alternative explanations for the mechanisms governing psychological processes—for instance, the previously-mentioned hypothesis of the conservation of emotional momentum (Frijda, 1992; Smedslund, 1992)—which are otherwise not possible with the AR(1) model with Gaussian innovations. Furthermore, the extensions of these models may be used for different kinds of time series, such as those with inherently quantitative discrete variables, either ordinal or categorical (see, e.g., Biswas et al., 2014; Pegram, 1980; Weiß, 2020), and connect them to the greater body of literature on dynamical processes, importantly, state-space models (Davis & Dunsmuir, 2016), Markov chains (Joe et al., 2016), and generalized linear models (Fokianos et al., 2016).

The current study may be improved and extended in a few ways. Since the association between the autoregressive parameter of the AR(1) model and person characteristics has been the core topic of interest in inertia research, in this paper, we only studied univariate time series. Furthermore, we took a very specific simulation strategy, namely, sampling the autoregressive parameters of individuals independent from their means, thus making the individual differences in means (and skewness) uncorrelated with the individual differences in the autoregressive parameter. This decision was made based on practical considerations, importantly, to minimize the degrees of freedom (and thus the number of conditions) in our simulation design. Finally, we only considered three alternative DGMs (and for the last model, we considered a very specific marginal distribution). Thus, future research may extend our study by investigating the effect of skewness and the floor effect on cross-lagged effects in bivariate and multivariate VAR(1) models, considering other simulation strategies (e.g., sampling the autoregressive parameters such that they have a fixed, non-zero correlation with the means), and exploring other (multivariate) non-Gaussian time series models as data-generating mechanisms.

In this paper, we only estimated the multilevel AR(1) model based on the assumption of normally distributed residuals at level 1 and 2. The reason for this was that the statistical software commonly used by psychological researchers does not yet include the non-Gaussian multilevel time series models that we used to generate the skewed data with. While these models have been largely overlooked in the psychological literature so far, they have been widely studied in other fields, such as hydrology or econometrics, for more than half a century. We believe there is merit to the use of these models in psychological research. The widespread use of them in modeling psychological time series requires a body of literature that is more accessible to empirical psychologists, and developing software packages capable of modeling such time series. Currently, the software packages dedicated to analyzing discrete-valued time series—for instance, the R packages *glarma* (Dunsmuir & Scott, 2015), *tscount* (Liboschik et al., 2017), *acp* (Vasileios, 2015), and *ZIM* (Yang et al., 2018)—are suited for a narrow set of count processes (that do not include, e.g., the parsimonious models we introduced) and can only analyze single subject (*N* = 1) time series. Thus, there is a need for developing software that would fill the gaps, importantly, multilevel modeling of a wider set of discrete-valued time series models, like the BinAR(1) or DAR(1) models. We hope that future research would address these issues and help popularize such models in psychological research.

## Article information

**Conflict of Interest Disclosures**: Each author signed a form for disclosure of potential conflicts of interest. No authors reported any financial or other conflicts of interest in relation to the work described.

**Ethical Principles**: The authors affirm having followed professional ethical guidelines in preparing this work. These guidelines include obtaining informed consent from human participants, maintaining ethical treatment and respect for the rights of human or animal participants, and ensuring the privacy of participants and their data, such as ensuring that individual participants cannot be identified in reported results or from publicly available original or archival data.

**Funding**: This work was supported by a Consolidator grant [grant agreement number 865468] from the European Research Council (ERC) under the European Union's Seventh Framework Programme [FP7/2007-2013].

**Role of the Funders/Sponsors**: None of the funders or sponsors of this research had any role in the design and conduct of the study; collection, management, analysis, and interpretation of data; preparation, review, or approval of the manuscript; or decision to submit the manuscript for publication.

**Acknowledgments**: The authors would like to thank the journal editor and the two anonymous reviewers for their comments on prior versions of this manuscript. The ideas and opinions expressed herein are those of the authors alone, and endorsement by the authors’ institutions or the European Research Council is not intended and should not be inferred.

## Reproducible code

All code used in this study to simulate and analyze the datasets and make the plots, as well as the results data files, can be accessed via the study's GitHub repository at https://github.com/psyguy/skewness-staging, and the simulation documentation can be found on https://psyguy.github.io/skewness-staging.

## Supplementary Material

Supplemental Material

## Figures and Tables

**Figure A2.**
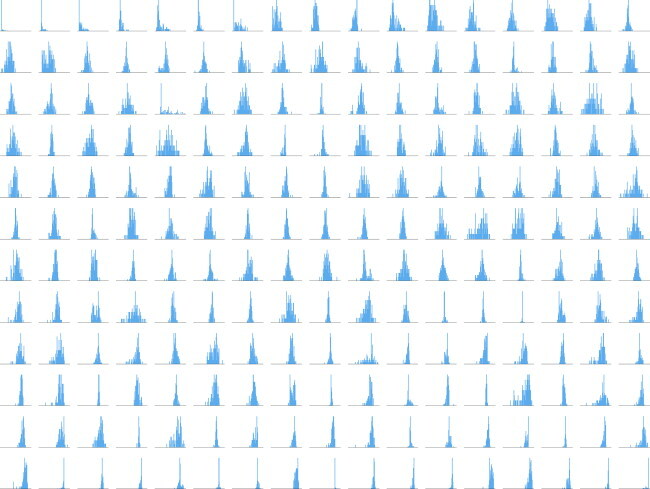
Individual histograms of PA scores ($X_{i,t}$) of individuals in the COGITO dataset, sorted by individual means (*μ_i_*).

**Figure A3.**
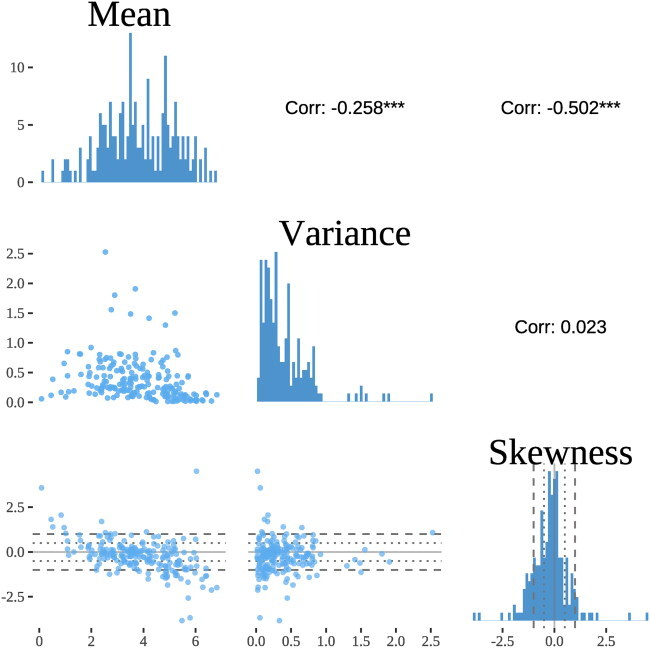
Histograms and pair-wise scatter plots of the individual summary statistics ($\mu_{i},\sigma_{i}^{2},\gamma_{i}$) of PA scores in the COGITO dataset, and the Pearson correlations between them (with *, **, and *** respectively denoting *p* < 0.05, *p* < 0.01, and *p* < 0.001). The dotted and dashed lines, respectively, mark the conventional thresholds of moderate (γ=±0.5) and high (γ=±1) skewness.

**Figure B3.**
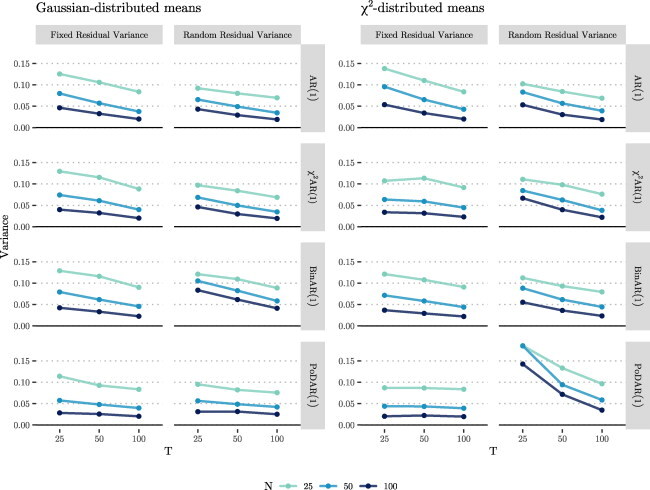
Estimation variance of the random effect correlation between *μ_i_* and $\phi_{i}.$

## Footnotes

1 Terluin et al. (2016) relied on previous findings that the “restriction of range” due to data selection based on an external criteria (Linn, 1968) can lead to the underestimation of the correlations among the variables affected by certain types of missing data mechanism (Wiberg & Sundström, 2019). However, it is unclear whether this phenomenon applies to the floor effect in affective time series, as the floor effect itself does not necessarily arise from a missingness mechanism (Falcaro et al., 2013; Oord & Ark, 1997).

2 The Supplemental Materials can be found on the Open Science Framework via https://osf.io/ngxdc.

3 It should be noted that the dissipation of water as explained here—as a linear function of *X_t_*_−1_—cannot be achieved by a simple physical system that is only under the influence of gravity. Importantly, the dissipation rate of a real water tank is proportional to the *square root* of the height of the liquid above the tap (see Safier, 2013), which cannot be modeled using an AR-type difference equation like the one in Equation 8. Thus, the water tank metaphor for the *χ*^2^AR(1) model should only be taken as a rough analogy rather than an accurate equivalent to a physical model.

4 The 95% CI (and not other value) as a decision rule is often used in Bayesian modeling to parallel the common decision threshold of α=0.05, a conventionally accepted threshold for Type-I error—the probability of mistakenly assuming a non-zero estimate for a parameter that, in reality, is zero—in null-hypothesis significant testing in the context of frequentist modeling. The overall convergence rate in our simulation was 99.75%. More specifically, among 144 conditions, there were 66 conditions that had a convergence rate of 100%, and 76 other conditions had convergence rates of at least 98.1%. The two remaining conditions had *χ*^2^-distributed means and were analyzed with the model with random residual variance, and belonged to the BinAR(1) model with N=100,T=100 and the PoDAR(1) model with N=100,T=25, which had convergence rates of 95.9 and 91.9%, respectively.

## Open Scholarship

This article has earned the Center for Open Science badge for Open Materials. The materials are openly accessible at https://osf.io/zf56v/.

## Appendix A. Detecting and characterizing the floor effect in empirical data

In the main text, we established the likely presence of the floor effect using visual inspection of person-specific histograms of empirical data (see Figure 2). Recall that the floor effect is defined by the co-occurrence of low mean, small variance, and a strong positive skewness of an individual’s response (i.e., marginal) distribution (Falcaro et al., 2013; Iachina & Bilenberg, 2012; Vermeersch et al., 2000). Since each of these sample statistics can be estimated directly from empirical data, we may also investigate the presence of the floor effect in an empirical dataset by computing the mean, variance and skewness value of each individual, and examining both their distributions and correlations across individuals. If the floor effect is present, we expect to see a positive correlation between the mean and the variance (low means coincide with low variance), negative correlations between the skewness and the mean (low means coincide with high positive skewness), and negative correlations between the skewness and the variance (low variance coincides with high positive skewness). Note that positive skewness values indicate clustering around the lower end of the response scale, while negative skewness values indicate clustering around the upper end of the rating scale, so we would also expect to observe positive skewness values in data with the floor effect.

In Figure A1 we show the relevant sample statistics and their correlations for the *distress* variable from the COGITO dataset (Schmiedek et al., 2010). The diagonal panels show the distribution of individual means, variances and skewness values, lower off-diagonal panels show pairwise scatter plots of the summary statistics, and the upper panels show their Pearson correlations. It can be seen from Figure A1 that lower means of distress very often coincide with smaller variability (leading to a positive correlation between mean and variances) and more asymmetry (leading to a negative correlation between mean and skewness), which is characteristic of data with a floor effect. The lower left panel of this figure (the scatter plot of skewness given mean) shows that most of the individuals have very low means and very high skewnesses, indicating that the floor effect in individuals (at level 1) brings about skewness in means (at level 2).

The same methodology can be applied to investigate the presence of a possible *ceiling effect*, the phenomenon whereby responses cluster near the top end of a rating scale. In the ceiling effect we would expect to see high negative skewness values coinciding with high means (in contrast to the floor effect, which is defined by low means), producing a negative correlation between skewness and mean. We would also expect a negative correlation between mean and variance (distributions with higher means have lower variances) and a positive correlation between skewness and variance. In Figure A2 we show the individual histograms of the Positive Affect (PA) from the COGITO dataset (Schmiedek et al., 2010), which is the unweighted average of 10 positive emotion items. It can be seen that some individuals are characterized by the floor effect, and some with the ceiling effect. Figure A3 shows the distribution of summary statistics of PA. We observe that the distribution of means is more symmetrical (upper left panel); however, as the histogram of skewnesses shows (lower right panel), some individuals have either positively or negatively skewed responses (notice that many of the skewnesses are either more than 1 or less than −1, typically used as thresholds for high positive and negative skewness). We observe a negative correlation between mean and skewness, and mean and variance, which likely indicates that more individuals are characterized by the ceiling effect for this variable.

## Appendix B. Additional notes on the simulation study

### B.1 Credibility intervals and the Type-I error rates

As discussed in the results section, increasing *T* reduces bias, RMSE, and Type-I error rates. However, an increase in *N* only decreases bias and RMSE, but leads to an increase in Type-I error rates. To study the reason for this behavior, we look at the confidence intervals of the estimated correlation between the estimated mean and autoregression at level 2. Figure B1 shows the point estimates of the correlation (white) and the corresponding 95% CIs for the BinAR(1) model with normally distributed means at level 2 for different *N*s and *T*s, either estimated with a model with fixed or random residual variance. The CIs are ordered based on the corresponding point estimates. As we can see, by an increase in *N* or *T* the point estimates, on average, get closer to zero (consequently reducing bias). The CIs also shrink when *N* or *T* increases, and the effect of *N* on this shrinkage is more noticeable. Given that by increasing *N* CIs shrink faster than the decrease of bias, for larger sample sizes, fewer CIs cover zero, which leads to an increase in Type-I error rates. As we can see, in the model with fixed residual variance, most of the errors fall below zero, and in the model with random residual variance, there are more errors, most of which, again, are below zero.

We also look at a similar plot for the PoDAR(1) model with *χ*^2^-distributed level-2 means in Figure B2. Here we observe that increasing *N* or *T* shrinks the CIs, and the shrinkage is faster when *N* increases. Compared to Figure B1, we observe that there are more errors for this DGM; the model with fixed residual variance has only negative Type-I errors, whereas the model with random residual variance has both positive and negative errors, though the number of positive ones is more than the negative ones.

### B.2 Estimation variance

In the main text, we presented Type-I error rates, bias, and RMSE in our estimates. Here, we present estimation variance which is another commonly used measure for the quality of estimators that quantifies the precision of the estimates (or how variable they are). The estimation variance contributes to the total estimation error (quantified by RMSE) *via* $\text{RMSE}=\sqrt{{\text{Bias}}^{2}+\text{Variance}}.$

We calculated the empirical estimation variance directly by calculating the variance of the estimated correlations in each condition, which are shown in Figure B3. As we see, increasing *N* or *T* reduced variance in all cases, regardless of the modeling technique, and the effect of the former was stronger. The modeling technique had an effect on the estimation variance; except for the AR(1) model (and *N* = 25 in other models), the estimation variance was always higher if a model with random residual variance (which is more complex than the model with fixed residual variance) was used. By comparing the estimation variance with the bias in the estimated correlations (Figure 9), we notice the bias–variance tradeoff: A more complex model may decrease the absolute value of the estimation bias, but it comes at the cost of increasing the variability in the estimates.
