# Supplementary material for: Skewness and Staging: Does the Floor Effect Induce Bias in Multilevel AR(1) Models?
Source: Multivariate Behav Res. 2023 Dec 31;59(2):289–319. doi: 10.1080/00273171.2023.2254769 (PMC11152569; doi:10.1080/00273171.2023.2254769)
Supplement: Supplemental Material [file HMBR_A_2254769_SM9514.pdf]

SUPPLEMENTAL MATERIALS

Skewness and staging: Does the floor effect induce bias in multilevel AR(1) models?

M. M. Haqiqatkhah[a], O. Ryan[a], and E. L. Hamaker[a]

[a]Department of Methodology and Statistics, Faculty of Social and Behavioural Sciences, Utrecht University

ARTICLE HISTORY
Compiled November 19, 2022
This version has not been peer reviewed yet.

In this document, we discuss, in details, the dynamic properties (importantly, the autocorrelation function) and the marginal properties (distribution, mean, variance, and skewness) of each of the data generating models introduced in the paper (Haqiqatkhah et al., 2022) and provide their derivations. Furthermore, for the last two model, we also demonstrate the equivalent Markov models. In order to give an impression of the marginal distributions of the time series generated by each DGM, we provide person histograms and distributions of summary statistics of the first simulated datasets (with $N = 100$ and $T = 100$) of the AR(1), χ^2AR(1), BinAR(1), and PoDAR(1) models, respectively, in Figures S1, S2, S4, and S5. In each figure, the upper panel belongs to the condition with normally distributed level-2 means, and the lower panel belongs to the condition where person means were drawn from a χ^2 distribution. See Appendix A for interpretations.

1. The AR(1) model

1.1. Marginal properties

We first derive the exact analytical formulae for a finite time series of length T, then generalize them to the asymptotic case, that is, for infinitely long time series ($T \to \infty$).

1.1.1. Marginal mean

To derive the marginal distribution of the AR(1) process we first need to find its mean, which is the expected value of X_T. To do so, we start by rewriting X_T based on its two previous values X_{T-1}, X_{T-2}:

CONTACT M. M. Haqiqatkhah. Email: m.h.haqiqatkhah@uu.nl. Address: Department of Methodology and Statistics, Faculty of Social and Behavioural Sciences, Utrecht University, P.O. Box 80140, 3508 TC, Utrecht, The Netherlands.

$$
\begin{aligned}
X_T &= c + \phi X_{T-1} + \epsilon_T \\
&= c + \phi(c + \phi X_{T-2} + \epsilon_{T-1}) + \epsilon_T \\
&= c + \phi c + \phi^2 X_{T-2} + \phi\epsilon_{T-1} + \epsilon_T,
\end{aligned}
\tag{S1}
$$

and then iteratively repeat the above step of substituting the previous value in the equation for $X_{T-2}, X_{T-3}, \ldots X_1$, which results in

$$
\begin{aligned}
X_T &= c + \sum_{i=1}^{T} \phi^i c + (\prod_{i=1}^{T} \phi^i) X_1 + \sum_{i=1}^{T-1} \phi^i \epsilon_i + \epsilon_T \\
&= \phi^0 c + \sum_{i=1}^{T} \phi^i c + (\prod_{i=1}^{T} \phi^i) X_1 + \sum_{i=1}^{T-1} \phi^i \epsilon_i + \phi^0 \epsilon_T \\
&= \sum_{i=0}^{T} \phi^i c + \phi^{1+2+3+\ldots+T} X_1 + \sum_{i=0}^{T} \phi^i \epsilon_i \\
&= \frac{c(1-\phi^T)}{1-\phi} + \phi^{T(T+1)/2} X_1 + \sum_{i=0}^{T} \phi^i \epsilon_i,
\end{aligned}
\tag{S2}
$$

in which the first element is the sum of a geometric sequence of length t with the starting term c and common ratio ϕ. To get the mean of the time series, we calculate the expected value of X_T, and given that the Gaussian innovation term has a mean of zero ($E[\epsilon_t] = 0$), we have

$$
\begin{aligned}
E[X_T] &= \frac{c(1-\phi^T)}{1-\phi} + \phi^{T(T+1)/2} E[X_1] + \sum_{i=0}^{T} \phi^i E[\epsilon_i] \\
&= \frac{c(1-\phi^T)}{1-\phi} + \phi^{T(T+1)/2} E[X_1] + \sum_{i=0}^{T} \phi^i 0 \\
&= \frac{c}{1-\phi}(1-\phi^T) + \phi^{T(T+1)/2} E[X_1].
\end{aligned}
\tag{S3}
$$

For an infinitely long sequence, given the limited range of ϕ ($|\phi| < 1$), the marginal expectation of the AR(1) process becomes its marginal mean—also referred to as its *stationary mean*—that is

$$
\begin{aligned}
\mu &= E[X_t] \\
&= \lim_{T\to\infty} E[X_T] \\
&= \frac{c}{1-\phi},
\end{aligned}
\tag{S4}
$$

which is finite and time-invariant (proving mean stationarity), and is equal to the expression in Equation 3.

1.1.2. Marginal distribution

To derive the marginal distribution of the AR(1) process, similar to Equation 6, we should first center it around its marginal expectation:

$$\begin{aligned}\tilde{X}_T &= X_T - E[X_T] \\ &= \phi\tilde{X}_{T-1} + \epsilon_T.\end{aligned} \tag{S5}$$

Furthermore, as we know, if two independent Gaussian random variables Y_a and Y_b are normally distributed with mean zero and common variance σ_Y^2 (i.e., $Y_a, Y_b \sim \mathcal{N}(0, \sigma_Y^2)$), the distribution of their weighted sum $Y_{sum} = aY_a + bY_2$ would again be Gaussian and is characterized by

$$Y_{sum} = \mathcal{N}(0, [a^2 + b^2]\sigma_Y^2) \tag{S6}$$

By generalizing this for $\tilde{X}_T, \tilde{X}_{T-1}, \dots \tilde{X}_1$, and following the same logic as of Equation S2, we will have

$$\begin{aligned}\tilde{X}_T &= (\prod_{i=1}^{T})\phi^i\tilde{X}_1 + \sum_{i=1}^{T}\phi^i\epsilon_i + \epsilon_T \\ &= \phi^{T(T+1)/2}\tilde{X}_1 + \sum_{i=0}^{T}\phi^i\epsilon_i \\ &\sim \mathcal{N}\Big(0, \phi^{T(T+1)/2}\sigma_{X_1}^2 + \sum_{i=0}^{T}(\phi^2)^i\sigma_\epsilon^2\Big) \\ &\sim \mathcal{N}\Big(0, \frac{\sigma_\epsilon^2}{1-\phi^2}(1-\phi^{2T}) + \phi^{T(T+1)/2}\sigma_{X_1}^2\Big).\end{aligned} \tag{S7}$$

By adding the expected value of X_T (Equation S3) to $\tilde{X}_T$, the marginal distribution will be

$$\begin{aligned}X_T &= E[X_T] + \tilde{X}_T \\ &\sim \mathcal{N}\Big(\frac{c}{1-\phi}(1-\phi^T) + \phi^{T(T+1)/2}E[X_1], \\ &\qquad \frac{\sigma_\epsilon^2}{1-\phi^2}(1-\phi^{2T}) + \phi^{T(T+1)/2}\sigma_{X_1}^2\Big).\end{aligned} \tag{S8}$$

Finally, if the sequence is infinitely long ($t \to \infty$), given that $|\phi| < 1$, the stationary distribution of X_t becomes

$$X_T \sim \mathcal{N}(\frac{c}{1-\phi}, \frac{\sigma_\epsilon^2}{1-\phi^2}), \tag{S9}$$

which is equal to the expression in Equation 4, and entails that the variance is $\sigma^2 =$

$\sigma_\epsilon^2/(1-\phi^2)$ and the skewness is zero ($\gamma = 0$). Given that the variance of the AR(1) model converged to a a finite, time-independent value, we may again conclude stationarity of variance.

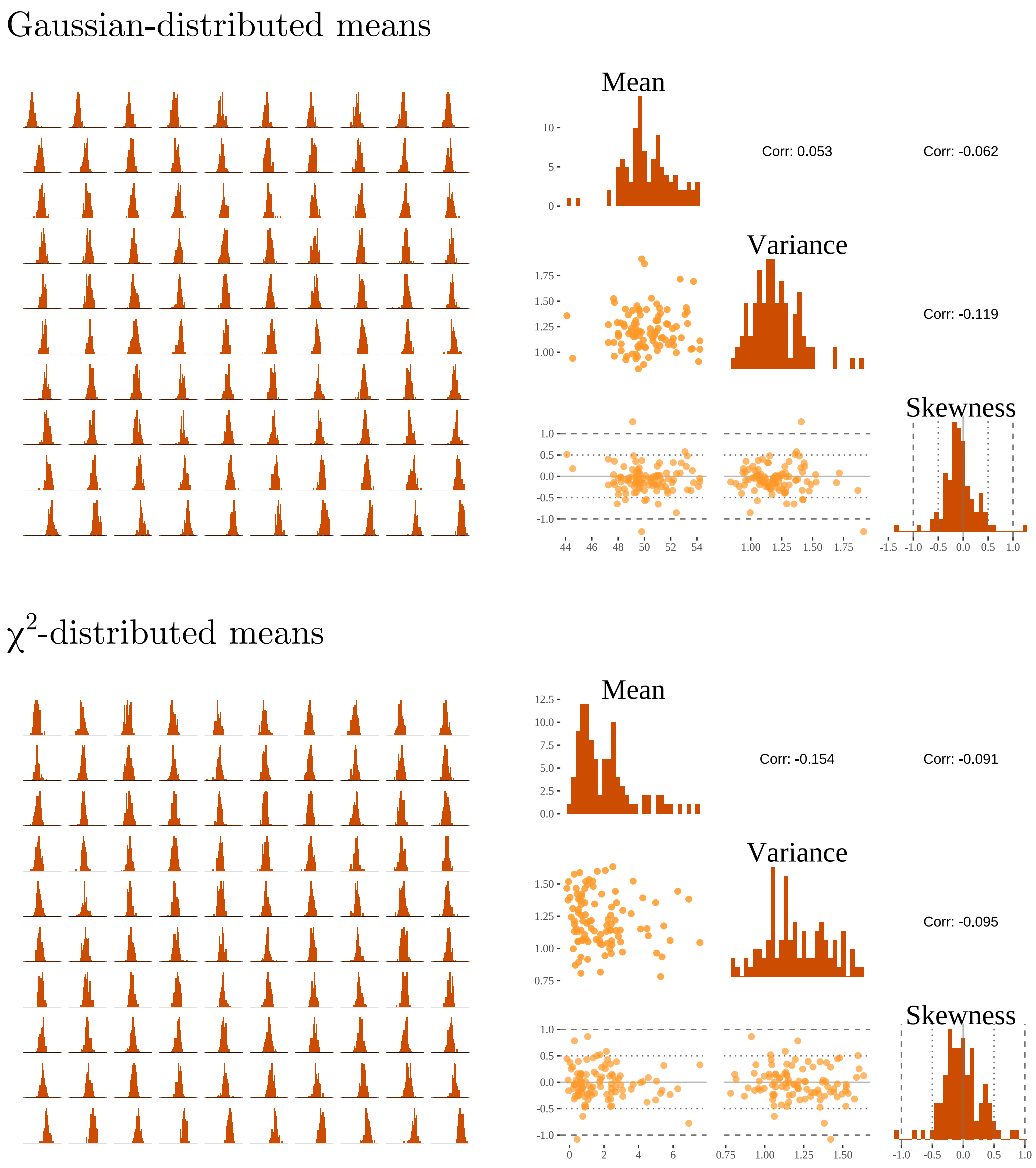

Figure S1. Individual histograms ($X_{i,t}$) (left) and the histograms and pair-wise scatter plots of the individual summary statistics ($\mu_i, \sigma_i^2, \gamma_i$) and the Pearson correlations between them (with *, **, and *** respectively denoting $p < .05$, $p < .01$, and $p < .001$) (right) of datasets generated by the **AR(1) model**, with level-2 means sampled from a Gaussian (top) or χ^2 (bottom) distribution. The dotted and dashed lines, respectively, mark the conventional thresholds of moderate ($\gamma = \pm 0.5$) and high ($\gamma = \pm 1$) skewness.

1.2. Dynamic properties

1.2.1. Autocorrelation function

To derive the ACF of the AR(1) model, we first calculate its autocovariance function, and then transform it to ACF by dividing it by the variance (gunes, 2019). We calculate AFC asymptotically (for infinitely long time series) and make use of the stationarity assumption of the AR(1) model. Let $\zeta(l)$ be the lag-l autocovariance of X_t, which entails, $\zeta(1) = Cov(X_t, X_{t-1})$ which means that $\zeta(0)_t = Cov(X_t, X_t) = Var(X_t) = \sigma^2$. We start by calculating $\zeta(1)$ via

$$\begin{aligned}\zeta(1) &= Cov(X_t, X_{t-1})\\ &= Cov(c + \phi X_{t-1} + \epsilon_t, X_{t-1})\\ &= Cov(c, X_{t-1}) + \phi Cov(X_{t-1}, X_{t-1}) + Cov(\epsilon_t, X_{t-1})\\ &= 0 + \phi\zeta(0) + 0\\ &= \phi\zeta(0),\end{aligned} \tag{S10}$$

in which $Cov(c, X_{t-1}) = 0$ because the covariance between a random variable and a constant is always zero, and $Cov(\epsilon_t, X_{t-1}) = 0$ because ϵ_t, by definition, is independent of X_{t-1}. Similarly, for $l = 2$ we have

$$\begin{aligned}\zeta(2) &= Cov(X_t, X_{t-2})\\ &= Cov(c + \phi X_{t-1} + \epsilon_t, X_{t-2})\\ &= Cov(c, X_{t-2}) + \phi Cov(X_{t-1}, X_{t-2}) + Cov(\epsilon_t, X_{t-2})\\ &= 0 + \phi Cov(X_t, X_{t-1}) + 0\\ &= \phi\zeta(1)\\ &= \phi^2\zeta(0).\end{aligned} \tag{S11}$$

By iteratively repeating the steps taken in Equations S10 and S11 we reach to the general formula of

$$\zeta(l) = \phi^l\zeta(0). \tag{S12}$$

The autocorrelation function of AR(1) can thus be calculated by dividing $\zeta(l)$ by the variance of X_t (i.e., $\zeta(0)$, which yields $\rho(l) = \phi^l$ for $l \geq 0$ (Equation 5), which shows an exponential decay.

2. The χ^2AR(1) model

The χ^2AR(1) process is a special case of the AR(1) process with gamma-distributed residuals, as a χ^2 distribution with ν degrees of freedom is equivalent to a gamma distribution with shape parameter $\alpha = \nu/2$ and scale parameter $\lambda = 2$, that is, $\chi^2(\nu) \sim \Gamma(\nu/2, 2)$. Thus, here we prove the properties for the general case where $a_t \sim \Gamma(\alpha, \lambda)$ and include an intercept in the model. We call such process the ΓAR(1) model which has the following form:

$$X_t = c + \phi X_{t-1} + a_t, \quad a_t \sim \Gamma(\alpha, \lambda). \tag{S13}$$

Including the intercept c makes the physical interpretation of the model harder, and it has been ignored in the literature because it does not have practical applications. However, we keep it in the model to derive more general formulae and make it more comparable to the AR(1) model. As we will see, c only appears in the marginal mean of the ΓAR(1) model and only changes the location of the marginal distribution. In this section, instead of deriving the expressions for a finite time series and then calculating their asymptotic limits, we make use of the stationarity assumption for $|\phi| < 1$—importantly, that the moments of the time series do not change over time (Tufto, 2021), thus they asymptotically converge to a specific value—which may be proven by taking the same steps in the previous section to derive the asymptotic properties of the AR(1) model.

2.1. Marginal properties

To derive the marginal properties of a ΓAR(1) model write X_t recursively based on its previous values $X_{t-2}, X_{t-3}, \ldots X_1$, following the steps taken in Equation S2. However, given that the weighted sum of gamma-distributed random variables does not have a closed-form distribution (Di Salvo, 2008), it is not possible to derive an analytical expression for the marginal distribution of the ΓAR(1) model (Tiku et al., 1999). Thus, we only derive the relevant moments of the process, namely, mean, variance, and skewness.

2.1.1. Marginal mean

We calculate the stationary mean of the ΓAR(1) process using the Law of Total Expectation—which states that the expected value of a random variable A is equal to the expected value of its expectation given another random variable B, that is, $E[A] = E[E[A|B]]$—with X_{t-1}, and make use of Equation S26:

$$\begin{aligned}
\mu &= E[X_t] \\
&= E\Big[E[X_t|X_{t-1}]\Big] \\
&= E\Big[E[c + \phi X_{t-1} + a_t|X_{t-1}]\Big] \\
&= E\Big[c + \phi X_{t-1} + E[a_t]\Big] \\
&= E[c + \phi X_{t-1} + \alpha\lambda] \\
&= c + \phi E[X_{t-1}] + \alpha\lambda \\
&= c + \phi E[X_t] + \alpha\lambda \\
&= c + \phi\mu + \alpha\lambda,
\end{aligned} \tag{S14}$$

thus,

$$\mu = E[X_t] = \frac{c + \alpha\lambda}{1 - \phi}. \tag{S15}$$

Substituting $c = 0$, $\alpha = \nu/2$, and $\lambda = 2$ in Equation S15 yields the marginal mean of the χ^2AR(1) model of Equation 10.

2.1.2. Marginal variance

In a similar manner, to calculate the marginal variance, we make use of the Law of Total Variance—which determines the variance of a random variable A based on another random variable B via $Var(A) = Var(E[A|B]) + E[Var(A|B)]$—with X_{t-1}:

$$\begin{aligned}\sigma^2 &= Var(X_t) \\ &= Var\Big(\underbrace{E[X_t|X_{t-1}]}_{\mathbb{I}}\Big) + E\Big[\underbrace{Var(X_t|X_{t-1})}_{\mathbb{III}}\Big],\end{aligned} \tag{S16}$$

in which $\mathbb{I}$ is the conditional expectation of X_t, which is equal to $c+\phi X_{t-1}+\alpha\lambda$, and $\mathbb{III}$ is the conditional variance of the process, which is equal to the variance of a_t, because

$$\begin{aligned}Var(X_t|X_{t-1}) &= E\Big[\big(X_t - E[X_t|X_{t-1}]\big)^2\Big|X_{t-1}\Big] \\ &= E\Big[\big(c - \phi X_{t-1} + a_t - c - \phi X_{t-1} - E[a_t]\big)^2\Big|X_{t-1}\Big] \\ &= E\Big[\big(a_t - E[a_t]\big)^2\Big] \\ &= Var(a_t) \\ &= \alpha\lambda^2.\end{aligned} \tag{S17}$$

Thus, Equation S16 simplifies to

$$\begin{aligned}\sigma^2 &= Var(X_t) \\ &= Var(c + \phi X_{t-1} + \alpha\lambda) + E[\alpha\lambda^2] \\ &= Var(X_{t-1}) + \alpha\lambda^2 \\ &= Var(X_t) + \alpha\lambda^2 \\ &= \sigma^2 + \alpha\lambda^2,\end{aligned} \tag{S18}$$

which yields

$$\sigma^2 = Var(X_t) = \frac{\alpha\lambda^2}{1-\phi^2}. \tag{S19}$$

Substituting $\alpha = \nu/2$, and $\lambda = 2$ in Equation S19 yields the marginal variance of the χ^2AR(1) model of Equation 11.

2.1.3. Marginal skewness

Following Tufto (2021), we make use of the Law of Total Cumulance (Brillinger, 1969) to calculate the stationary third moment (κ_3) of X_t and make use of the results from earlier:

$$\begin{aligned}\kappa_3 &= \mu_3(X_t) \\ &= E\Big[\underbrace{\mu_3(X_t|X_{t-1})}_{\mathbb{I}}\Big] + \underbrace{\mu_3\Big(E[X_t|X_{t-1}]\Big)}_{\mathbb{II}} + \\ &\underbrace{3Cov\Big(E[X_t|X_{t-1}], Var(X_t|X_{t-1})\Big)}_{\mathbb{III}}.\end{aligned} \tag{S20}$$

Following similar steps as in Equation S17, we may show that $X_t|X_{t-1}$ and a_t have the same third central moment ($\mu_3(X_t|X_{t-1}) = \mu_3(a_t)$). Thus, to calculate $\mathbb{I}$, we may make use of the cumulant generating function of a_t. Specifically, given that the moment generating function of the gamma-distributed a_t is $M_a(s) = (1 - \lambda s)^{-\alpha}$ (Krishnamoorthy, 2016), its cumulant generating function is $K_a(s) = \ln\big(M_a(s)\big) = -\alpha \ln(1 - \lambda s)$. Thus we can calculate $\mu_3(X_t|X_{t-1})$ by taking the third derivative of $K_a(s)$ at zero:

$$\begin{aligned}\mu_3(a_t) &= \frac{\mathrm{d}^3 K_a(s)}{\mathrm{d}s^3}\bigg|_{s=0} \\ &= \frac{\mathrm{d}^3\big[-\alpha\ln(1-\lambda s)\big]}{\mathrm{d}s^3}\bigg|_{s=0} \\ &= \frac{-2\alpha\lambda^3}{(\lambda s - 1)^3}\bigg|_{s=0} \\ &= 2\alpha\lambda^3.\end{aligned} \tag{S21}$$

By substituting the conditional expectation of X_t in $\mathbb{II}$ and using the stationarity assumption, we have

$$\begin{aligned}\mu_3(X_t|X_{t-1}) &= \mu_3(c + \phi X_{t-1} + \alpha\lambda) \\ &= \phi^3 \mu_3(X_{t-1}) \\ &= \phi^3 \mu_3(X_t) \\ &= \phi^3 \kappa_3.\end{aligned} \tag{S22}$$

Then, given that the conditional variance of X_t is a constant ($Var(X_t|X_{t-1}) = \alpha\lambda^2$; Equation S17), its covariance with $E[X_t|X_{t-1}]$ (i.e., $\mathbb{III}$) is zero. Consequently, Equation S20 becomes

$$\begin{aligned}\kappa_3 &= E[2\alpha\lambda^3] + \phi^3\kappa_3 + 0 \\ &= 2\alpha\lambda^3 + \phi^3\kappa_3,\end{aligned} \tag{S23}$$

which, solving for κ_3, yields

$$\kappa_3 = \frac{2\alpha\lambda^3}{1 - \phi^3}. \tag{S24}$$

Finally, the skewness of X_t can be calculated by dividing κ_3 by σ^3, which yields

$$\gamma = \frac{\kappa_3}{\sigma^3} = \frac{2(1-\phi^2)^{3/2}}{\sqrt{\alpha}(1-\phi^3)}. \tag{S25}$$

By substituting $\alpha = \nu/2$ in Equation S25, we get to the marginal skewness of the χ^2AR(1) model of Equation 12.

2.2. *Dynamic properties*

2.2.1. *Conditional expectation*

The conditional expectation of the ΓAR(1) model, which is its deterministic part given the previous observation, may be easily derived via

$$\begin{aligned} E[X_t|X_{t-1}] &= E[c + \phi X_{t-1} + a_t|X_{t-1}] \\ &= c + \phi E[X_{t-1}|X_{t-1}] + E[a_t|X_{t-1}] \\ &= c + \phi X_{t-1} + E[a_t] \\ &= c + \phi X_{t-1} + \alpha\lambda, \end{aligned} \tag{S26}$$

which, for $c = 0$, $\alpha = \nu/2$, and $\lambda = 2$, is equal to the conditional expectation of the χ^2AR(1) model in Equation 9.

2.2.2. *Autocorrelation function*

To calculate the ACF of the ΓAR(1) model, like that of the AR(1) model, we should derive its lag-l autocovariance function $\zeta(l)$, for which we start by $\zeta(1)$:

$$\begin{aligned} \zeta(1) &= Cov(X_t, X_{t-1}) \\ &= Cov(c + \phi X_{t-1} + a_t, X_{t-1}) \\ &= Cov(c, X_{t-1}) + \phi Cov(X_{t-1}, X_{t-1}) + Cov(a_t, X_{t-1}) \\ &= 0 + \phi Cov(X_t, X_t) + 0 \\ &= \phi\zeta(0). \end{aligned} \tag{S27}$$

As we can see, the lag-1 autocovariance of the ΓAR(1) model is identical to that of the AR(1) model and independent of the residual distribution. Thus we may follow the same steps taken for the AR(1) process, the ACF of ΓAR(1) model will be $\rho(l) = \phi^l$ for $l \geq 0$.

3. The BinAR(1) model

3.1. *Marginal properties*

It can be shown that if the first observation of a BinAR(1) process is taken from a binomial distribution with success probability θ and size k (i.e., $X_1 \sim Binom(\theta, k)$),

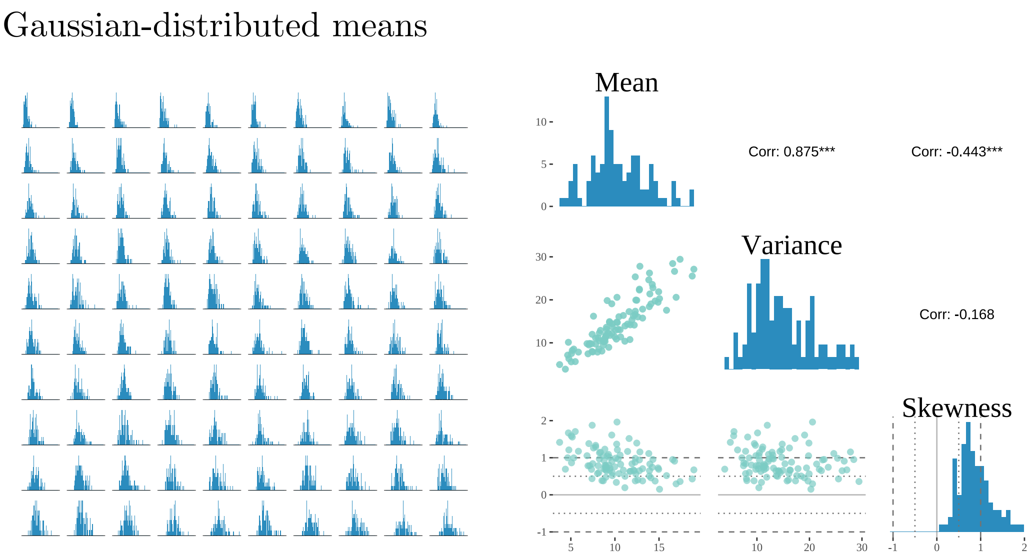

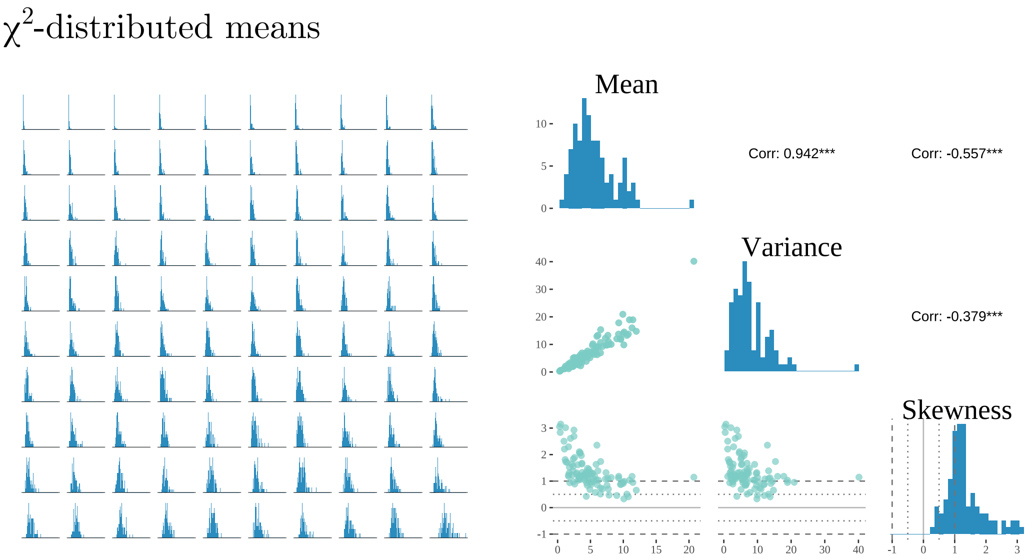

Figure S2. Individual histograms ($X_{i,t}$) (left) and the histograms and pair-wise scatter plots of the individual summary statistics ($\mu_i, \sigma_i^2, \gamma_i$) and the Pearson correlations between them (with *, **, and *** respectively denoting $p < .05$, $p < .01$, and $p < .001$) (right) of datasets generated by the $\boldsymbol{\chi^2}$**AR(1) model**, with level-2 means sampled from a Gaussian (top) or χ^2 (bottom) distribution. The dotted and dashed lines, respectively, mark the conventional thresholds of moderate ($\gamma = \pm 0.5$) and high ($\gamma = \pm 1$) skewness.

later observations of the process follow the same distribution (i.e., $X_t \sim Binom(\theta, k)$ for $t = 2, 3, \dots$) (see, e.g., Al-Osh & Alzaid, 1991). One should, however, show how θ is related to the survival and revival probabilities α and β. To do so, McKenzie (1985) constructs the BinAR(1) model starting with its marginal distribution, which is considered to be $X_t \sim Binom(\theta, k)$, and argues that for any survival probability $0 \leq \alpha \leq 1$, a BinAR(1) model of a form similar to Equation 13[1] exists if we define $\beta = (1-\alpha)\theta/(1-\theta)$, which results in an autocorrelation parameter of $\phi = \alpha - \beta$. To assure that $0 \leq \beta \leq 1$ (such that it may be considered a probability), there must be a constraint on the values of θ given ϕ (Weiß & Kim, 2013), which is

$$\max\left\{\frac{-\theta}{1-\theta}, \frac{1-\theta}{-\theta}\right\} \leq \phi \leq 1. \tag{S28}$$

Given this constraint, we may plot the admissible area of the BinAR(1) model in the ϕ–θ parameter plane, which is shown in Figure S3. In this figure, α and β corresponding to any permissible pairs of ϕ and θ are plotted with contour lines, and the plot is colored based on the marginal skewness (with conventional thresholds of $|\gamma| \leq 0.5$, $0.5 < |\gamma| \leq 1$, and $|\gamma| > 1$) resulting from the θ parameter. As we can see, α and β can freely take any values from 0 to 1, and as long as the autoregressive parameter is non-negative ($\phi \geq 0$), θ can also take any values from 0 to 1. Given that the BinAR(1) model has a binomial distribution with $X_t \sim Binom(\theta, k)$ wherein $\theta = \frac{\beta}{1-(\alpha-\beta)}$, its mean, variance, and skewness are easily computed using Equations 16–18.

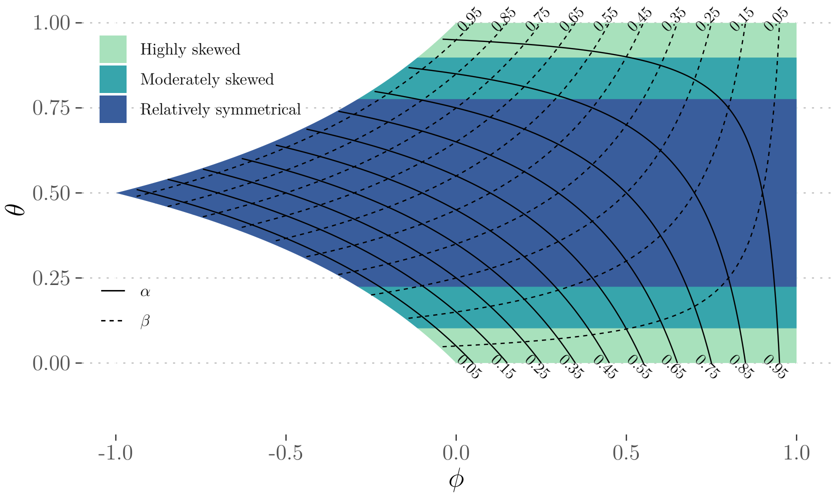

Figure S3. The admissible ranges of the BinAR(1) model parameters with $k = 7$. The contour lines show values of α and β corresponding to any given admissible θ and ϕ pairs. The area is shaded based on the marginal skewness implied by θ, with conventional thresholds of $|\gamma| \leq 0.5$, $0.5 < |\gamma| \leq 1$, and $|\gamma| > 1$, respectively for negligible, moderate, and high skewness.

[1]It should be noted that, in the literature cited so far, the *binomial thinning* operator (Steutel & van Harn, 1979) has been used in characterizing the BinAR(1) model—which we refrained from using this operator in this paper to reduce complexity—makes the derivation of its properties easier.

3.2. Dynamic properties

3.2.1. Equivalent Markov model

As shown by Al-Osh and Alzaid (1991) and Weiß (2009), the BinAR(1) model can also be expressed as a special, parsimonious case of a first-order Markov model: The $0-k$ integer values of the BinAR(1) process can be thought of as $k+1$ distinct *states*, such that the probability of being in a specific state at any given time depends solely on the state the person was in at the previous occasion and a 1-step-ahead transition probability. Particularly, the transition probability of going from $X_{t-1}=u$ to $X_t=v$ (for $u,v=0,...,k$) in the BinAR(1) model with parameters α and β can be expressed as

$$\begin{aligned} p_{v|u} =& P(X_t=v|X_{t-1}=u) \\ =& \sum_{m=max\{0,u+v-k\}}^{min\{u,v\}} \binom{u}{m}\binom{k-u}{v-m} \alpha^m(1-\alpha)^{u-m}\beta^{v-m}(1-\beta)^{k-u+m-v}. \end{aligned} \tag{S29}$$

To provide an impression of what the matrix of transition probabilities looks like for the BinAR(1) models, we include the 2×2 transition matrix T_1 for a model with $k=1$, and the 3×3 matrix T_2 for the model with $k=2$, that are

$$\begin{aligned} T_1 =& \begin{array}{c} \\ 0 \\ 1 \end{array} \begin{array}{c} \begin{array}{cc} 0 & 1 \end{array} \\ \begin{bmatrix} 1-\beta & 1-\alpha \\ \beta & \alpha \end{bmatrix} \end{array}, \\ T_2 =& \begin{array}{c} \\ 0 \\ 1 \\ 2 \end{array} \begin{array}{c} \begin{array}{ccc} 0 & 1 & 2 \end{array} \\ \begin{bmatrix} (1-\beta)^2 & (1-\alpha)(1-\beta) & (1-\alpha)^2 \\ 2\beta(1-\beta) & \alpha(1-\beta)+(1-\alpha)\beta & 2\alpha(1-\alpha) \\ \beta^2 & \alpha\beta & \alpha^2 \end{bmatrix} \end{array}. \end{aligned} \tag{S30}$$

These show that the elements of the matrix with transition probabilities are functions only two parameters, namely, α and β.

3.2.2. Autocorrelation function

Following the derivations of the previous models, let $\zeta(l)$ denote the lag-l autocovariance of X_t, thus $\zeta(1)=Cov(X_t,X_{t-1})$ and $\zeta(0)=Cov(X_t,X_t)=Var(X_t)$. We start by calculating the lag-1 autocovariance of X_t, that is

$$\begin{aligned} \zeta(1) &= Cov(X_t, X_{t-1}) \\ &= Cov(S_t+R_t, X_{t-1}) \\ &= Cov(S_t, X_{t-1}) + Cov(R_t, X_{t-1}). \end{aligned} \tag{S31}$$

To calculate the first term, we use the definition of covariance

$$Cov(S_t, X_{t-1}) = \underbrace{E[S_t X_{t-1}]}_{\mathbb{I}} - \underbrace{E[S_t]E[X_{t-1}]}_{\mathbb{III}}, \tag{S32}$$

and use the Law of Total Expectation (Ziddletwix, 2021) to calculate $\mathbb{I}$, which yields

$$\begin{aligned} E[S_t X_{t-1}] &= E\Big[E[S_t X_{t-1}|X_{t-1}]\Big] \\ &= E\Big[X_{t-1}E[S_t|X_{t-1}]\Big] \\ &= E[X_{t-1}X_{t-1}\alpha] \\ &= \alpha E[X_{t-1}^2]. \end{aligned} \tag{S33}$$

Note that given S_t has a binomial distribution with size X_{t-1} and probability of success α, its expectation given X_{t-1} is equal to the mean of a binomially distributed random variable, which is $X_{t-1}\alpha$. To calculate $\mathbb{III}$, we use the Law of Total Expectation for S_t, which entails

$$\begin{aligned} E[S_t] &= E\Big[E[S_t|X_{t-1}]\Big] \\ &= E[\alpha X_{t-1}] \\ &= \alpha E[X_{t-1}] \\ \implies E[S_t]E[X_{t-1}] &= \alpha E[X_{t-1}]^2. \end{aligned} \tag{S34}$$

Putting Equations S32–S34 together, we have

$$\begin{aligned} Cov(S_t, X_{t-1}) &= \alpha E[X_{t-1}^2] - \alpha E[X_{t-1}]^2 \\ &= \alpha(E[X_{t-1}^2] - E[X_{t-1}]^2) \\ &= \alpha Var(X_{t-1}) \\ &= \alpha Var(X_t). \end{aligned} \tag{S35}$$

Similarly, one can easily show $Cov(R_t, X_{t-1}) = \beta Var(k - X_t) = \beta Var(X_t)$. Thus

$$\begin{aligned} \zeta(1) &= (\alpha - \beta)Var(X_t) \\ &= (\alpha - \beta)\zeta(0). \end{aligned} \tag{S36}$$

Following the steps leading to Equation S12, it easily follows that $\zeta(2) = (\alpha - \beta)\zeta(1) = (\alpha - \beta)^2\zeta(0)$, and by extension, $\zeta(l) = (\alpha - \beta)^l\zeta(0)$ for $l \geq 0$ (Al-Osh & Alzaid, 1987). Thus the autocorrelation function of the BinAR(1) model is $\rho(l) = \zeta(l)/\zeta(0) = (\alpha - \beta)^l$ for $l \geq 0$.

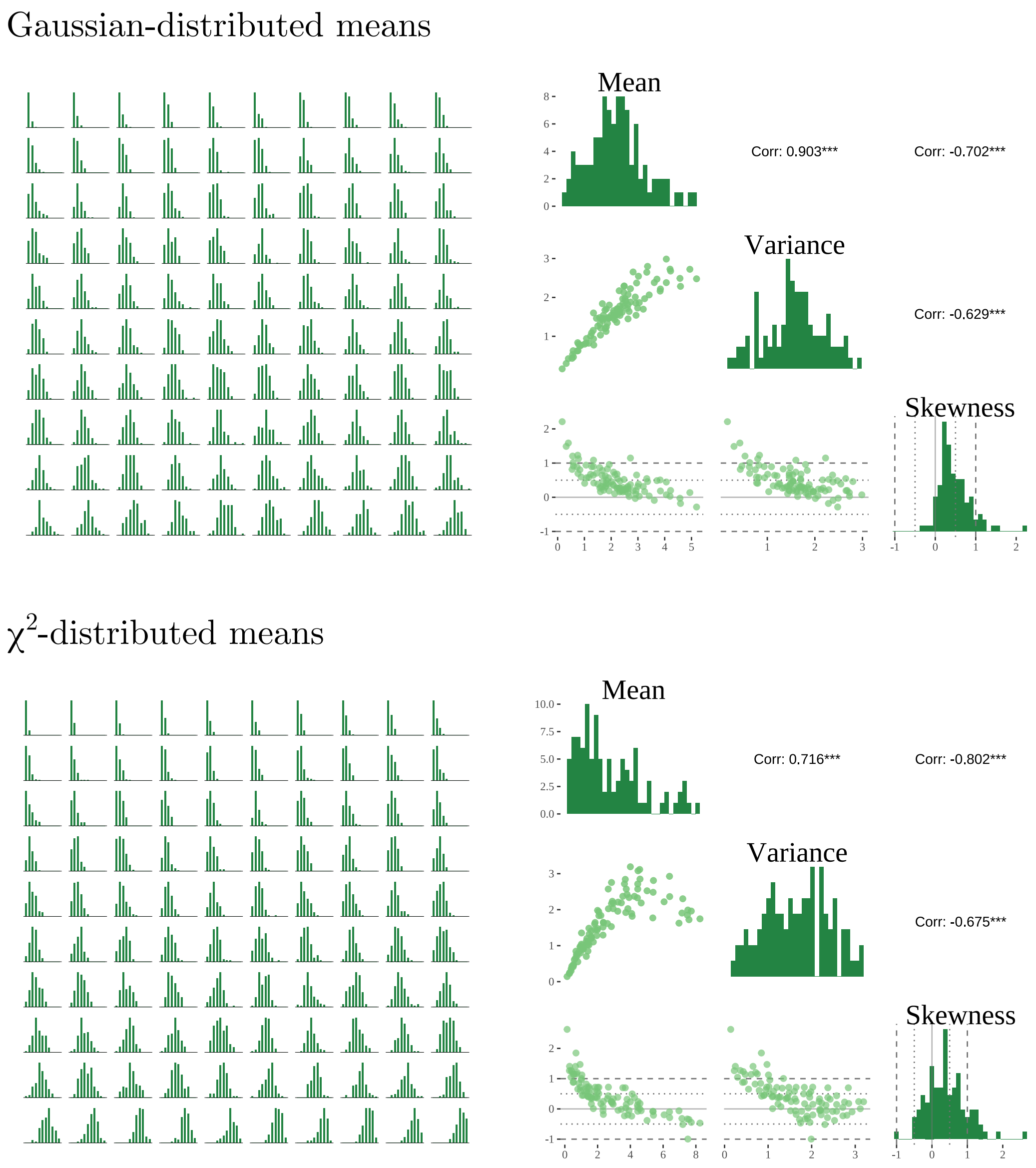

Figure S4. Individual histograms $(X_{i,t})$ (left) and the histograms and pair-wise scatter plots of the individual summary statistics $(\mu_i, \sigma_i^2, \gamma_i)$ and the Pearson correlations between them (with *, **, and *** respectively denoting $p < .05$, $p < .01$, and $p < .001$) (right) of datasets generated by the **BinAR(1) model**, with level-2 means sampled from a Gaussian (top) or χ^2 (bottom) distribution. The dotted and dashed lines, respectively, mark the conventional thresholds of moderate $(\gamma = \pm 0.5)$ and high $(\gamma = \pm 1)$ skewness.

4. The PoDAR(1) model

As explained in the main text, the properties of the DAR(1) model are independent of its marginal distribution—whether bounded (like the binomial and beta-binomial distributions) or unbounded (like Poisson, geometric, or negative binomial distributions)—as long as it is discrete-valued.[2] Thus, in this section, we discuss the properties of this model in its general form (with an arbitrary marginal distribution of $X_t \sim \Pi$). However, when presenting its equivalent Markov model, we also introduce an upper-bounded version of the PoDAR(1) model with right-truncated Poisson marginal distribution, which is more suitable for scales with an upper bound and its Markov model can be expressed in a transition matrix.

4.1. Marginal properties

The DAR(1) model is characterized by the τ and Π, which are the persistence probability and the distribution of Z_t. The main marginal property of the DAR(1) model is that it has the same marginal distribution as Z_t, that is, $X_t \sim Z_t \sim \Pi$, and it is independent of τ. To do so, let us assume that the first observation of the time series followed the distribution of Z_t, that is, $X_1 \sim \Pi$. Given the formulation of Equation 20, we may see that X_2, a random variable, is a mixture of two other random variables, X_1 and Z_2:

$$X_2 = P_2 X_1 + (1 - P_2) Z_2. \tag{S37}$$

We know that if A and B are two random variables with probability mass functions $f_A(i)$ and $f_B(i)$, their weighted sum $C = w_A A + w_B B$ (if $w_A, w_B \geq 0$ and $w_A + w_B = 1$) has a density which is a mixture of A and B and has a probability mass function given by $f_C(i) = w_A f_A(i) + w_B f_B(i)$. In case A and B have the same distribution ($A \sim B$, thus $f_A(i) = f_B(i)$) it follows that $f_C(i) = (w_A + w_B) f_A(i) = f_A(i)$, which entails that C has the same distribution as A ($C \sim A$).

Applying this to Equation S37, given that $X_1 \sim Z_2 \sim \Pi$ and the weights (P_2 and $1 - P_2$) always add up to 1 regardless of the value of τ, we may conclude that $X_2 \sim \Pi$. By repeating this for other measurement occasions $t = 3, 4, \ldots$, we can show that $X_t \sim \Pi$. Given that in the PoDAR(1) model $Z_t \sim Poisson(\lambda)$, its marginal mean, variance, and skewness are readily calculated from the formulas of the Poisson distribution (Equation 23).

4.2. Dynamic properties

4.2.1. Equivalent Markov model

It can be easily shown that the DAR(1) model of Equation 19 is a special case of a first-order Markov process by enumerating the different ways the person may have a value of v at the measurement occasion t ($X_t = v$) (Jacobs & Lewis, 1978). Assuming that at the previous occasion, the person had a value of u ($X_{t-1} = u$), the person may have the same value of distress at occasion t ($v = u$) in two ways: Either (a) no disruption takes place between $t - 1$ and t ($P_t = 1$, with probability τ) thus the person keeps the

[2] It is worth noting that in the literature the DAR(1) model has been primarily used for bounded distributions. However, as we discussed, the model definition does not imply such restriction.

same level of distress ($X_t = X_{t-1}$); or (b) a disruption happens ($P_t = 0$ with probability $1-\tau$), but with a probability of π_u the person ends up experiencing the same level of distress on occasion t ($X_t = Z_t = u$). Because P_t is independent of X_{t-1} and Z_t, the total probability of keeping the same level of distress is $\tau + (1-\tau)\pi_v$. Conversely, the only way that a person can have a different level of distress on occasion t is that both a disruption happens ($P_t = 0$ with probability $1-\tau$), and they experience another level of distress ($v \neq u$) with the probability π_v. Given the independencies, this probability will be $(1-\tau)\pi_v$. Consequently, the transition probability of going from u at $t-1$ to v at t, denoted by $p_{v|u}$, can be expressed as

$$p_{v|u} = P(X_t = v | X_{t-1} = u) = \begin{cases} (1-\tau)\pi_v, & \text{for } v \neq u \\ \tau + (1-\tau)\pi_v, & \text{for } v = u \end{cases}. \tag{S38}$$

In case Π is not upper-bounded, as in the PoDAR(1) model, the process will be a countable-state infinite Markov process (with countably infinite number of states $v = 0, 1, \ldots$), which is hard to express by a transition matrix. However, one may impose an upper bound of k for the process, for instance, on the grounds of substantive or measurement considerations. In that case, another marginal distribution can be used for Π, and the resulting DAR(1) model may be characterized by an ordinary lag-1 Markov transition matrix T, which can be written using Ω (a $k \times k$ matrix whose columns are Π), and I (identity matrix of size k) as follows (McKenzie, 2003):

$$T = \tau I + (1 - \tau\Omega) = \Omega - \tau(\Omega - I) \tag{S39}$$

By applying the Bayes' rule to Equation S39, one can easily show that the marginal distribution of the DAR(1) is equal to Π (and is independent of τ).

As an example, and following the rationale of using the Poisson distribution for stress units exposing the person at a constant rate of λ, we may use the right-truncated Poisson distribution (Suaiee, 2013) as an upper-bounded marginal distribution for Π, which has the following probability mass function:

$$\begin{aligned} \pi_{i_k} = P(X_t = i | i \leq k) &= \frac{\frac{\lambda^i e^{-\lambda}}{i!}}{\sum_{m=0}^{k} \frac{\lambda^m e^{-\lambda}}{m!}} \\ &= \frac{\lambda^i}{i! \sum_{m=0}^{k} \frac{\lambda^m}{m!}} \\ &= \psi_k \frac{\lambda^i}{i!}, \quad \text{with} \quad \psi_k = \left(\sum_{m=0}^{k} \frac{\lambda^m}{m!} \right)^{-1}. \end{aligned} \tag{S40}$$

There are closed form expressions for moments of the right-truncated Poisson distribution, however, they are quite complex (see, e.g., Suaiee, 2013, pp. 24-25). If k is much larger than λ—that is, the upper limit of the scale is chosen conservatively high—the term ψ_k in Equation S40, using power series expansion, can be approximated by $e^{-\lambda}$, thus

$$\pi_{i_k} = \psi_k \frac{\lambda^i}{i!} \approx \frac{\lambda^i e^{-\lambda}}{i!} \quad \text{for } k >> \lambda, \tag{S41}$$

which is similar to the probability mass function of the non-truncated Poisson distribution (Equation 22). As a result, when $k >> \lambda$,[3] we may conveniently approximate mean, variance, and skewness of the right-truncated PoDAR(1) with

$$\begin{aligned} E[X_t] &= \mu_{\text{trunPoDAR(1)}} \approx \lambda, \\ Var[X_t] &= \sigma^2_{\text{trunPoDAR}} \approx \lambda, \text{ and} \\ Skewness[X_t] &= \gamma_{\text{trunPoDAR(1)}} \approx \sqrt{1/\lambda}. \end{aligned} \tag{S42}$$

To provide an impression of what the matrix of transition probabilities resulting from Equation S39 looks like, here we show the transition matrix of a right-truncated PoDAR(1) model with $k = 7$, which is fully characterized by λ and τ:

$$T_3 = \begin{array}{c} \\ 0 \\ 1 \\ 2 \\ \vdots \\ 7 \end{array} \begin{array}{c} \begin{array}{ccccc} 0 & 1 & 2 & \dots & 7 \end{array} \\ \begin{bmatrix} \tau + (1-\tau)\psi_7 & (1-\tau)\psi_7 & (1-\tau)\psi_7 & \dots & (1-\tau)\psi_7 \\ (1-\tau)\psi_7\lambda & \tau + (1-\tau)\psi_7\lambda & (1-\tau)\psi_7\lambda & \dots & (1-\tau)\psi_7\lambda \\ (1-\tau)\psi_7\frac{\lambda^2}{2} & (1-\tau)\psi_7\frac{\lambda^2}{2} & \tau + (1-\tau)\psi_7\frac{\lambda^2}{2} & \dots & (1-\tau)\psi_7\frac{\lambda^2}{2} \\ \vdots & \vdots & \vdots & \ddots & \vdots \\ (1-\tau)\psi_7\frac{\lambda^7}{5040} & (1-\tau)\psi_7\frac{\lambda^7}{5040} & (1-\tau)\psi_7\frac{\lambda^7}{5040} & \dots & \tau + (1-\tau)\psi_7\frac{\lambda^7}{5040} \end{bmatrix} \end{array},$$

$$\text{where } \psi_7 = \left(\sum_{m=0}^{7} \frac{\lambda^m}{m!}\right)^{-1} \tag{S43}$$

4.2.2. Autocorrelation function

To calculate the ACF of the DAR(1) model, like those of the other models, we first derive the lag-l autocovariance function $\zeta(l)$. Using Equation 20, we start with $\zeta(1)$ and make use of the independencies between P_t, Z_t, and X_{t-1}:

[3]The difference between k and λ does not need to be so large. For instance, if on a $0-7$ Likert scale we have means that are no more than 4 ($\lambda \leq 4$, which was the case for 99% of all the measurements of negative items in the COGITO dataset), $0.946e^{-\lambda} < \psi_7 < e^{-\lambda}$, and if the person means on a $0-100$ scale are less than 75 ($\lambda \leq 75$, which would be the case for many affective time series), $0.998e^{-\lambda} < \psi_{100} < e^{-\lambda}$, both of which are very decent approximations.

$$
\begin{aligned}
\zeta(1) &= Cov(X_t, X_{t-1}) \\
&= Cov(P_t X_{t-1} + (1-P_t)Z_t, X_{t-1}) \\
&= Cov(P_t X_{t-1}, X_{t-1}) + Cov((1-P_t)Z_t, X_{t-1}) \\
&= E\Big[\big(P_t X_{t-1} - E[P_t X_{t-1}]\big)\big(X_{t-1} - E[X_{t-1}]\big)\Big] + 0 \\
&= E\Big[P_t X_{t-1}^2 - P_t X_{t-1} E[X_{t-1}] - E[P_t X_{t-1}] X_{t-1} + E[P_t X_{t-1}] E[X_{t-1}]\Big] \\
&= E[P_t X_{t-1}^2] - E[P_t X_{t-1} E[X_{t-1}]] - E[E[P_t X_{t-1}] X_{t-1}] + E[E[P_t X_{t-1}] E[X_{t-1}]] \\
&= E[P_t]E[X_{t-1}^2] - E[P_t]E[X_{t-1}]E[X_{t-1}] - E[P_t]E[X_{t-1}]E[X_{t-1}] + E[P_t]E[X_{t-1}]E[X_{t-1}] \\
&= E[P_t]\big(E[X_{t-1}^2] - E[X_{t-1}]^2\big) \\
&= \tau Var(X_{t-1}) \\
&= \tau Var(X_t) \\
&= \tau \zeta(0).
\end{aligned}
\tag{S44}
$$

This shows that the lag-1 autocovariance of the DAR(1) model is independent of marginal distribution Π and is identical to that of an AR(1) model with $\phi = \tau$. Like for other DGMs, we may follow the same steps taken for the AR(1) model to reach the ACF of the DAR(1) model, which is $\rho(l) = \tau^l$ for $l \geq 0$.

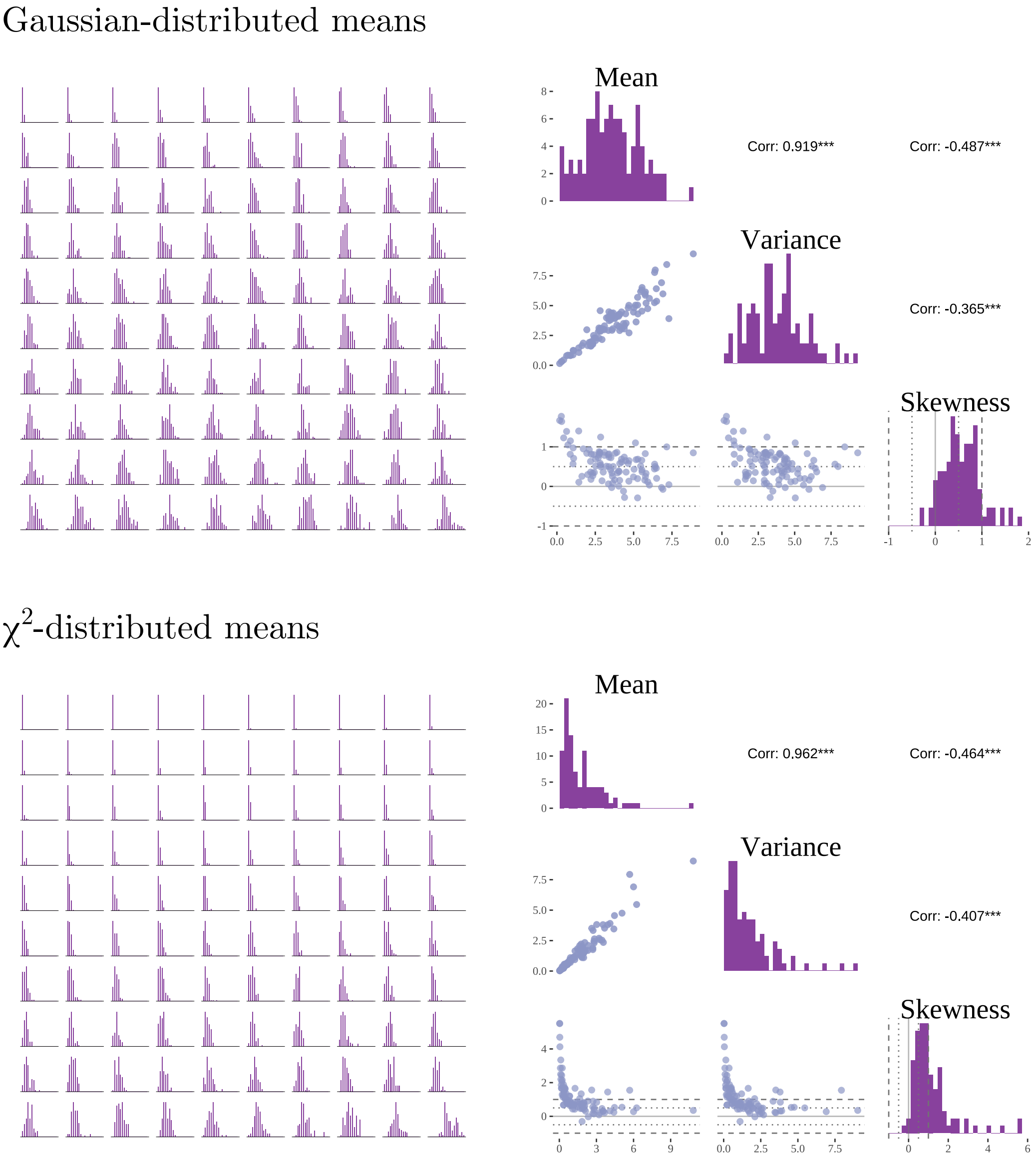

Figure S5. Individual histograms ($X_{i,t}$) (left) and the histograms and pair-wise scatter plots of the individual summary statistics ($\mu_i, \sigma_i^2, \gamma_i$) and the Pearson correlations between them (with *, **, and *** respectively denoting $p < .05$, $p < .01$, and $p < .001$) (right) of datasets generated by the **PoDAR(1) model**, with level-2 means sampled from a Gaussian (top) or χ^2 (bottom) distribution. The dotted and dashed lines, respectively, mark the conventional thresholds of moderate ($\gamma = \pm 0.5$) and high ($\gamma = \pm 1$) skewness.

References

Al-Osh, M. A., & Alzaid, A. A. (1987). First-Order Integer-Valued Autoregressive (INAR(1)) Process. *Journal of Time Series Analysis*, *8*(3), 261–275. https://doi.org/10.1111/j.1467-9892.1987.tb00438.x

Al-Osh, M. A., & Alzaid, A. A. (1991). Binomial autoregressive moving average models. *Communications in Statistics. Stochastic Models*, *7*(2), 261–282. https://doi.org/10.1080/15326349108807188

Brillinger, D. R. (1969). The calculation of cumulants via conditioning. *Annals of the Institute of Statistical Mathematics*, *21*(1), 215–218. https://doi.org/10.1007/BF02532246

Di Salvo, F. (2008). A characterization of the distribution of a weighted sum of gamma variables through multiple hypergeometric functions. *Integral Transforms and Special Functions*, *19*(8), 563–575. https://doi.org/10.1080/10652460802045258

gunes. (2019). Proof expression for the autocovariance function of AR(1).

Haqiqatkhah, M. M., Ryan, O., & Hamaker, E. L. (2022). Skewness and staging: Does the floor effect induce bias in multilevel AR(1) models? https://doi.org/10.31234/osf.io/myuvr

Jacobs, P. A., & Lewis, P. A. W. (1978). Discrete Time Series Generated by Mixtures. I: Correlational and Runs Properties. *Journal of the Royal Statistical Society. Series B (Methodological)*, *40*(1), 94–105.

Krishnamoorthy, K. (2016). *Handbook of statistical distributions with applications* OCLC: 957731970.

McKenzie, E. (1985). Some Simple Models for Discrete Variate Time Series. *Journal of the American Water Resources Association*, *21*(4), 645–650. https://doi.org/10.1111/j.1752-1688.1985.tb05379.x

McKenzie, E. (2003). Discrete variate time series. *Stochastic Processes: Modelling and Simulation* (pp. 573–606). Elsevier. https://doi.org/10.1016/S0169-7161(03)21018-X

Steutel, F. W., & van Harn, K. (1979). Discrete Analogues of Self-Decomposability and Stability. *The Annals of Probability*, *7*(5), 893–899.

Suaiee, A. M. A. (2013). *Double truncated poisson regression model with random effects* (Doctoral dissertation). University of Northern Colorado.

Tiku, M. L., Wong, W. K., & Bian, G. (1999). Time series models with asymmetric innovations. *Communications in Statistics - Theory and Methods*, *28*(6), 1331–1360. https://doi.org/10/fvj6df

Tufto, J. (2021). Moments (mean and skewness) of an AR(1) process with Chi2 or Gamma innovation distribution.

Weiß, C. H. (2009). Monitoring correlated processes with binomial marginals. *Journal of Applied Statistics*, *36*(4), 399–414. https://doi.org/10.1080/02664760802468803

Weiß, C. H., & Kim, H.-Y. (2013). Parameter estimation for binomial AR(1) models with applications in finance and industry. *Statistical Papers*, *54*(3), 563–590. https://doi.org/10.1007/s00362-012-0449-y

Ziddletwix. (2021). Covariance between a binomial random variable and its size (number of trials) (found in the context of binomial thinning).
